# Autophagy in MASLD: A Metabolic and Precision Medicine Perspective

**DOI:** 10.1111/liv.70816

**Published:** 2026-07-29

**Authors:** Alessandra Cazzaniga, Silvia Frigo, Alessandro Cherubini, Eniada Rrapaj, Luca Valenti

**Affiliations:** ^1^ Department of Pathophysiology and Transplantation Università degli Studi di Milano Milan Italy; ^2^ Precision Medicine Lab—Biological Resource Center, Transfusion Medicine Fondazione IRCCS Ca’ Granda Ospedale Maggiore Policlinico Milan Italy

**Keywords:** ATG7, cirrhosis, hepatocellular carcinoma, PNPLA3, steatosis

## Abstract

Metabolic dysfunction‐associated steatotic liver disease (MASLD) is the most common chronic liver condition worldwide and a major contributor to cirrhosis and hepatocellular carcinoma (HCC). While metabolic triggers such as obesity and insulin resistance are key drivers of MASLD, growing evidence has identified defects in intracellular quality control—namely impaired autophagy—as central mechanisms governing disease progression. Autophagy, including selective lipophagy and mitophagy, plays a crucial role in hepatic lipid turnover and mitochondrial homeostasis. In MASLD, disruption of these processes contributes to lipid accumulation and oxidative stress, leading to hepatocellular damage (ballooning), fibrogenesis, and HCC. Experimental studies linked impaired autophagic flux to liver injury, and emerging evidence from human genetics suggests that inter‐individual inherited variation influences MASLD susceptibility by impairing autophagy. Specifically, main genetic MASLD modifiers such as the p.I148M variant of Patatin‐like phospholipase domain‐containing protein 3 (*PNPLA3*) and loss‐of‐function and hypomorphic variants in autophagy‐related gene 7 (*ATG7*), a core autophagy gene, predispose to ballooning, fibrosis, and HCC. By outlining emerging therapies that restore autophagic flux and reduce steatosis, lipotoxicity, and fibrosis, we propose an integrated precision‐medicine model based on genetics and autophagy dynamics biomarkers, offering a new framework for personalized therapeutics.

## Key Points

- Impaired autophagy contributes to hepatocellular lipid accumulation, oxidative stress, and fibrogenesis in MASLD, linking hepatocyte stress with immune and endothelial activation.
- Autophagy has a dual role in HCC: tumour‐suppressive during early stages but tumour‐promoting in established cancers.
- MASLD genetic risk variants (*PNPLA3*, *ATG7*) impair autophagy and lipophagy, driving fibrogenesis and HCC.
- Approved therapeutics for fibrotic MASH combine metabolic improvement with autophagy enhancement.

**Abbreviations**
ABHD5: abhydrolase domain containing 5, lysophosphatidic acid acyltransferase
ACC: acetyl‐CoA carboxylase
AML12: alpha mouse liver 12
AMPK: 5′‐prime‐AMP‐activated protein kinase
APOB: apolipoprotein B100
ATG: autophagy‐related gene
BFAR: bifunctional apoptosis regulator
BH: ballooned hepatocytes
BNIP3: BCL2 interacting protein 3
CCL: C‐C motif chemokine ligand
cGAMP: cyclic guanosine monophosphate‐adenosine monophosphate
cGAS: cyclic GMP‐AMP
CHIP: carboxy‐terminus of Hsc70‐interacting protein
ChREBP: carbohydrate‐responsive element‐binding protein
CIDEC: cell death inducing DFFA like effector C
CMA: chaperone‐mediated autophagy
COL1A2: collagen type I alpha 2 chain
DAMPs: damage‐associated molecular Patterns
DGAT2: diacylglycerol O‐acyltransferase 2
DNL: de novo lipogenesis
ECM: extracellular matrix
EMT: endothelial‐to‐mesenchymal transition
ER: endoplasmic reticulum
ESCRTS: endosomal sorting complex required for transport
ETC: mitochondrial electron transport chain
FAK: focal adhesion kinase
FAO: fatty acid oxidation
FASN: SREBP‐1c and fatty acid synthase
FDA: food and drug administration
FFA: free fatty acids
FOXO1: Forkhead box protein O1
FUNDC1: FUN14 domain containing
GLP1: glucagon‐like protein‐1
GLP‐1RA: GLP1 receptor agonists
HCC: hepatocellular carcinoma
HDAC6: histone deacetylase 6
HDL: high‐density lipoprotein
HepG2 cell: hepatoma G2 cell
HFD: high‐fat diet
HIF‐1α: hypoxia inducible factor 1 subunit alpha
HPC: hepatic progenitor cells
HSC: hepatic stellate cells
HSC70: heat shock protein family A (Hsp70) member 8
HSL: hormone‐sensitive lipase
Huh‐7: human hepatoma cells
IFN‐γ: interferon‐gamma
IL: interleukin
IRF3: interferon regulatory factor 3
JNK: JUN N‐terminal Kinase
KC: Kupffer cells
KEAP1: Kelch like ECH associated protein‐1
LAMP2A: lysosome‐associated membrane protein type 2A
LC3B: microtubule‐associated protein 1 light chain 3 B
LD: lipid droplet
LKB1: liver kinase B1
LSEC: liver sinusoidal endothelial cells
MAP 1: MAPK phosphatase‐1
MASH: metabolic dysfunction‐associated steatohepatitis
MASLD: metabolic dysfunction‐associated steatotic liver disease
MDB: Mallory–Denk bodies
MDMs: monocyte‐derived macrophages
MT‐CYB: mitochondrial cytochrome b
mtDNA: mitochondrial DNA
mTORC: mechanistic target of rapamycin kinase
mtROS: mitochondrial ROS
NBR1: neighbour of BRCA1 gene 1
NF‐KB: nuclear factor kappa B
NLRP3: NLR family pyrin domain containing
NO: nitric oxide
NOTCH1: notch receptor 1
NRF2: NFE2 like BZIP transcription factor 2
NSD2: nuclear receptor binding SET domain protein 2
OMM: outer mitochondrial membrane
p62/SQSTM1: sequestosome 1
PARL: presenilin associated rhomboid like
PE: phosphatidylethanolamine
PGC1α: PPARG coactivator 1 alpha
PI3K: phosphatidylinositol 3‐kinase
PI3P: phosphatidylinositol 3‐phosphate
PINK1: PTEN induced kinase 1
PLIN: periplin
PNPLA: patatin‐like phospholipase domain‐containing protein
PPARα: peroxisome proliferator‐activated receptor gamma
Rab7: Ras‐associated GTP‐binding protein 7
ROCK: Rho associated coiled‐coil containing protein kinase
ROS: reactive oxygen species
S100A11: S100 calcium binding protein A11
SIRT: Sirtuin
SNARE: soluble N‐ethylmaleimide‐sensitive factor attachment protein receptor
SREBP: sterol regulatory element binding transcription factor 2
STING: synthase‐stimulator of interferon genes
SXT17: syntaxin 17
T2D: type 2 diabetes
TAG: triacylglycerols
TFEB: transcription factor EB
TFGβ: transforming growth factor‐beta
TG: triglycerides
THR‐β: thyroid hormone receptor‐β
TM6SF2: transmembrane 6 superfamily Member 2
TNFα: tumour necrosis factor alpha
TOM: translocase of the outer membrane
TRIM32: human tripartite motif family of proteins 32
Ub: ubiquitin
ULK1: Unc‐51‐like autophagy‐activating kinase
VAMP 8: vesicle associated membrane protein 8
VCAM‐1: vascular cell adhesion molecule—1
VLDL: very low‐density lipoproteins
VPS34: phosphatidylinositol 3‐kinase catalytic subunit type 3
YAP: yes‐associated protein
4‐PBA: 4‐phenylbutyrate
αSMA: alpha smooth muscle actin

## 1 An Overview on Autophagy

Autophagy (‘self‐eating’) is a highly conserved self‐degradative process that maintains cellular homeostasis by removal of misfolded proteins, damaged organelles, nucleic acids and lipids through lysosome‐mediated pathways, supporting cell survival, differentiation and metabolism [1, 2].

Key autophagy‐related genes (ATG) were identified by Yoshinori Ohsumi in 1993 elucidating autophagy's fundamental role in physiology and disease—a breakthrough that later earned him the Nobel Prize [2, 3]. Three major autophagic pathways have been described, distinguished by their cargo delivery mechanisms: (i) macro‐autophagy that involves autophagosome formation (Figure S1A); (ii) micro‐autophagy, which occurs through lysosomal membrane invagination to directly sequester cytosolic material (Figure S1B); (iii) chaperone‐mediated autophagy (CMA) that leads to selective protein degradation (Figure S1C) [4].

The lipidation of microtubule‐associated protein 1 light chain 3 B (LC3B) driven by ATG7, is crucial for autophagosome formation and fusion with lysosome, regulating substrate selection and recruitment in LC3B‐associated both macro‐ and micro‐autophagy [5]. ATG7 deacetylation enhances its interaction with ATG3, promoting both canonical and non‐canonical autophagy [5]. The multifunctional receptor protein 62 (p62)/sequestome 1 (SQSTM1) supports this process by directing ubiquitinated proteins to lysosomes, maintaining protein quality control, when proteasome is overwhelmed [6].

Macro‐ and micro‐autophagy can be non‐selective by randomly sequestering cytoplasmic cargo [7], or selective by targeting damaged organelles, as in lipophagy and mitophagy [8]. Lipophagy, regulated by perilipins (PLINs), controls lipid droplets (LD) mobilization and degradation under nutrient deprivation (Figure S2A). Similarly, mitophagy removes damaged mitochondria via PTEN Induced Kinase 1 (PINK1)/Parkin signalling, preserving energy balance and mitochondrial quality (Figure S2B) [9, 10].

Given its central role in lipid and organelle homeostasis, autophagy has emerged as a key regulator of metabolic dysfunction‐associated steatotic liver disease (MASLD), a complex disease triggered by metabolic cues in the presence of a favourable genetic *milieu* [11].

MASLD affects one‐third of the global population and is defined by hepatic steatosis (hepatic fat ≥ 5.5% of liver mass) in the presence of cardiometabolic risk factors such as obesity, type 2 diabetes (T2D), arterial hypertension, hypertriglyceridemia, or low HDL cholesterol, after the exclusion of harmful alcohol intake and other secondary causes. MASLD spans from simple steatosis to metabolic dysfunction‐associated steatohepatitis (MASH), which can progress to fibrosis, cirrhosis, and hepatocellular carcinoma (HCC) [12, 13]. Key genetic drivers of MASLD include *Patatin‐like* phospholipase domain‐containing protein‐3 (*PNPLA3*) and Transmembrane 6 superfamily member 2 (*TM6SF2*) variants that disrupt lipid handling, and rare *ATG7* mutations associated with worsened hepatocellular injury [14, 15, 16, 17, 18]. Hepatic steatosis in MASLD arises from LD accumulation in hepatocytes, mainly under the form of triglycerides (TAG) and cholesteryl esters that store fatty acids (FFAs) and free cholesterol [13]. Lipid overload impairs mitochondrial fatty acid oxidation (FAO), leading to increased reactive oxygen species (ROS) production and mitochondrial dysfunction. This, in turn, disrupts autophagy and promotes steatosis, inflammation, and progression to steatohepatitis and fibrosis [19, 20]. This review provides evidence that impaired autophagy facilitates MASLD progression and the transition to HCC, highlighting the role of human genetics and the potential of integrating genetic and autophagic biomarkers to guide precision‐medicine strategies.

## 2 Autophagy in Liver Physiology and MASLD

**FIGURE 1:**
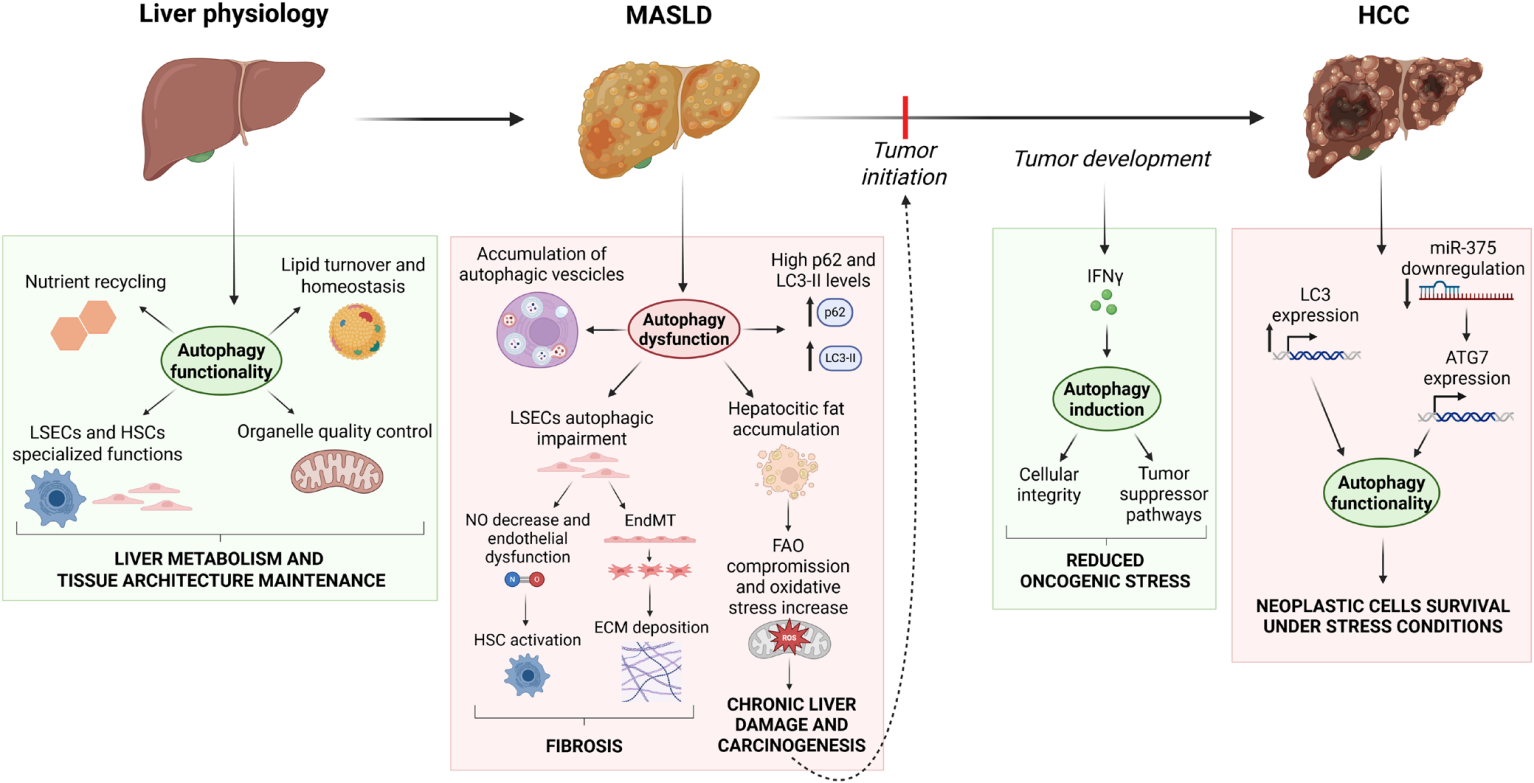
Autophagy involvement in liver physiology, MASLD and HCC. Autophagy activation in normal liver physiology ensures metabolic regulation and maintains tissue architecture. In MASLD patients, the intracellular accumulation of autophagic vesicles and the higher levels of p62 and LC3‐II indicate a dysfunctional autophagic process, responsible for endothelial dysfunction and HSC activation, leading to fibrosis, as well as for hepatocytic fat accumulation, consequently causing an increase in oxidative stress. The resulting chronic liver damage favours carcinogenesis: In the first stage of tumour development, autophagy plays the role of tumour suppressor, while later on, in HCC advanced stage, autophagy ensures neoplastic cells' survival and tumour progression. ATG, autophagy‐related gene; ECM, extracellular matrix; EMT, endothelial‐to‐mesenchymal transition; FAO, fatty acid oxidation; HCC, hepatocellular carcinoma; HSC, Hepatic Stellate Cells; IFN‐γ, Interferon‐gamma; LC3, Microtubule‐associated protein 1 light chain 3 B; LSEC, Liver Sinusoidal Endothelial cells; MASLD, Metabolic dysfunction‐associated steatotic liver disease; NO, nitric oxide; p62, Sequestosome‐1; ROS, reactive oxygen species.

The liver plays a central role in regulating the metabolism of macronutrients and in the detoxification of xenobiotics. Hepatocytes, the main parenchymal cells, rely on basal autophagy for nutrient recycling, lipid turnover, and organelle quality control. Non‐parenchymal cells, namely liver sinusoidal endothelial cells (LSECs), hepatic stellate cells (HSCs), and Kupffer cells (KCs), also depend on autophagy to maintain their specialized functions, support tissue architecture, and coordinate responses to injury. In LSECs, autophagy maintains fenestrations and supports anti‐fibrotic, anti‐thrombotic, and vasodilatory functions (Figure 1) [21]. Impaired autophagic flux reduces nitric oxide (NO) bioavailability and increases oxidative stress, leading to endothelial dysfunction, activation of HSCs, endothelial‐to‐mesenchymal transition (EMT), and extracellular matrix (ECM) deposition, ultimately promoting liver fibrosis (Figure 1) [21, 22].

The role of autophagy in HSCs is multifaceted and context dependent. Autophagy activation in HSCs favours fibrogenesis, whereas its inhibition reduces collagen production and cellular activation [23, 24]. Genetic models corroborate these conclusions: HSC‐specific deletion of *ATG7* prevents LD degradation and HSC activation upon injury, reducing fibrosis [25]. An overall impairment of the autophagy flux in the liver, especially in hepatocytes, has been robustly demonstrated during MASLD (Table 1). Hepatocytes regulate systemic lipid homeostasis by taking up or synthesizing FFAs, converting them into TAGs stored in LD, and balancing their fate between very low‐density lipoprotein (VLDL) secretion and FAO in mitochondria for energy production. When intracellular fat becomes excessive and the ability to secrete VLDL is saturated, FAO becomes the main route for FFA disposal. This maximal activation of FAO increases ROS generation and causes mitochondrial damage [20]. Ultimately, in experimental models, impairment of autophagy results in heightened predisposition to hepatic inflammation, hepatocellular injury, and fibrogenesis.

**TABLE 1:** Autophagy in MASLD.

Pathway/Markers	Molecular change	Mechanistic role in autophagy	Functional impact in MASLD	Model evidence	Clinical adaptability	References
AMPK1	↑ Activation	AMPK inhibition and autophagy suppression	MASH progression	Human + murine model	*Low/Moderate*: Invasive analysis needed (liver biopsy). While detectable via IHC, assessing its active (phosphorylated) state is technically challenging and sensitive to sample processing delays	[26]
ULK‐1, BECN‐1, LC3B	↓ Expression	Impaired autophagy initiation (ULK1 complex, phagophore formation)	Reduced autophagic flux → lipid accumulation, hepatocyte stress	Human liver sample	Low: invasive analysis needed (liver biopsy) and cellular isolation	[27, 28]
LC3B, p62 (SQSTM1)	↑ LC3‐II/LC3‐I ratio; ↑ p62 accumulation	Defective autophagosome formation and degradation	Impaired cargo clearance → lipotoxicity, inflammation	Murine MASH/HCC models	Moderate: Reliable histological marker, but requires fixed tissue (biopsy) and pathological expertise	[29]
ATG5, ATG7	↓ Expression/activity	Autophagosome elongation defect	Severe impairment of lipophagy → steatosis progression	Murine models	Low: invasive analysis needed (liver biopsy) and cellular isolation	[30]
NSD2/TFEB axis	↑NSD2 expression levels; ↓ TFEB expression level	Reduced autophagy gene expression and lysosomal biogenesis	Lipid accumulation → hepatic steatosis progression	Human MASLD liver tissue	Low: invasive analysis needed (liver biopsy) and cellular isolation	[31]
RUBICON	↑ Expression	Inhibits autophagosome–lysosome fusion	Autophagy blockade → inflammation, disease progression	Human + mouse MASH	Low: invasive analysis needed (liver biopsy) and cellular isolation	[32]
STX17, VAMP8	↓ Expression	Defective autophagosome–lysosome fusion	Accumulation of damaged organelles and lipids	Murine MASH models	Low: invasive analysis needed (liver biopsy) and cellular isolation	[33]
BNIP3	↓ Expression	Mitophagy deficiency	MASH and fibrosis progression	Human liver sample	Low: invasive analysis needed (liver biopsy) and cellular isolation	[27]
miR‐204‐3p	↓ Levels	Dysregulates ULK1/VPS34 signalling	Promotes HSC activation → fibrosis	Human MASLD tissues	Moderate/High: microRNAs are easily extracted from blood and quantified by qPCR	[34]
Cathepsin D	↑ Serum levels	Decrease lysosomal function (late‐stage autophagy)	Correlates with fibrosis severity; impaired degradation	Transcriptomic analysis in MASLD/MASH patients	High: easily measured in serum and analysed by ELISA	[35, 36]

*Note:* MKP1 is upregulated in NASH patients and mouse models. High levels of nuclear MKP1 prevent the cytosolic translocation of LKB1, thereby suppressing AMPK activation. This mechanistic block leads to impaired autophagy and increased hepatocyte death, significantly driving MASH progression [26]. Decreased expression of key autophagy initiators (ULK‐1, BECN‐1) and markers (ATG5, ATG7.LC3B, p62) reported in human and murine liver tissues [27, 28, 29, 30]; NSD2 overexpression in MASLD cohort epigenetically suppressed TFEB transcription, resulting in the inhibition of lysosome biogenesis, exacerbating the MASLD progression [31]. Rubicon overexpression, binding to the BECN‐1, inhibits autophagosome‐lysosome fusion and impairs autophagic flux [32]. Decreased expression of both STX17 and VAMP8 prevents autophagosome‐lysosome fusion and is correlated with MASLD progression [33]; downregulation of BNIP3 is observed in human liver biopsies, suggesting a mitophagy impairment [27]; Reduced levels of miR‐204‐3p—known to inhibit HSC activation and positively regulate autophagic flux via ULK1 and VPS34—were reported in MASLD/MASH patients [37]; Serum cathepsin D (CTSD) correlates with disease severity and improves FIB‐4 diagnostic accuracy [35, 36].

Abbreviations: AMPK, 5′‐AMP‐activated protein kinase catalytic; ATG, autophagy related gene; BECN1, Beclin1; LC3B, microtubule‐associated protein 1 light chain 3B; LKB1, liver kinase B1; MKP1, MAPK phosphatase‐1; NSD2, nuclear receptor binding SET domain protein 2; p62, Sequestosome 1; SXT17, Syntaxin 17; TFEB, Transcription Factor EB; ULK1, unc‐51 like autophagy activating kinase 1; VAMP 8, Vesicle Associated Membrane Protein 8; VPS34, Phosphatidylinositol 3‐Kinase Catalytic Subunit Type 3.

Notably, an accumulation of autophagic vesicles has been observed in liver biopsies from patients with chronic liver disease [35], suggesting disrupted autophagic flux. Consistently, patients with MASLD display a significant upregulation of cathepsin D, a key lysosomal protease, highlighting lysosomal dysfunction as a potential contributor to impaired autophagy and positioning cathepsin D as a promising biomarker for disease severity [35, 36].

In keeping, an early activation of autophagy followed by late‐phase characterized by p62 and LC3‐II accumulation has been observed in experimental models of MASLD, both in vivo and in vitro. This context dependent switch is driven by a dual defect in autophagy regulation. During the early stages of lipid overload, hepatocytes activate a protective autophagic response through mTORC1 downregulation, thereby promoting lipophagy and limiting lipotoxic damage [38]. Conversely, chronic fatty acid exposure induces aberrant mTORC1 reactivation, suppressing autophagy initiation and correlating with progressive steatosis [39, 40]. In parallel, autophagic flux is further impaired by Rubicon upregulation, which inhibits autophagosome–lysosome fusion [32, 41]. Consequently, interruption of autophagic flux leads to the accumulation of undegraded cargo, exacerbation of ER stress, hepatocyte apoptosis, and fibrogenesis [32, 39, 40, 41]. Interestingly, while the loss of autophagic efficiency drives hepatocyte injury, sustained autophagic activity in HSCs supports their activation and fuels fibrosis. Thus, the detrimental role of autophagy in late‐stage disease arises from its distinct and opposing effects across different hepatic cell populations [23, 24].

## 3 Lipophagy Dysfunction in MASLD

**FIGURE 2:**
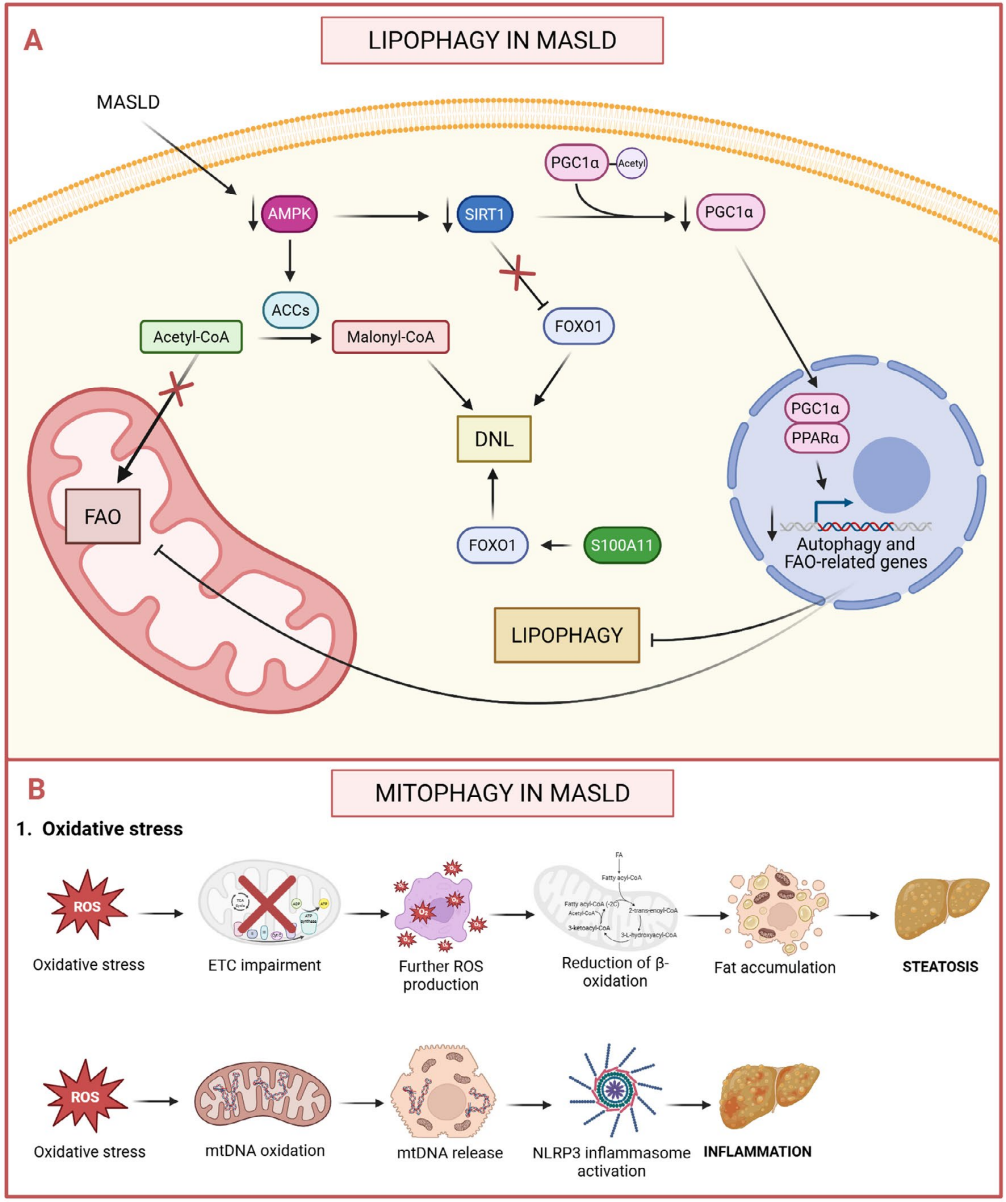
Lipophagy and mitophagy dysfunction in MASLD. (A) Lipid metabolism dysfunction in MASLD: The activation of AMPK inhibits the ACC enzymes, stopping the conversion of acetyl‐CoA, which accumulates and enhances FAO, to malonyl‐CoA, resulting in DNL inhibition. SIRT1, activated by AMPK, deacetylates PGC1α, which acts as coactivator of PPARα, leading to the upregulation of FAO‐related genes that regulate lipophagy. SIRT1 is also responsible for suppressing lipogenesis through inhibition of FOXO1. On the contrary, the newly discovered player S100A11, overexpressed in MASLD livers, correlates with FOXO1 activation, enhancing lipogenesis. (B) Mechanisms by which defective mitophagy promotes MASLD: 1. The increased oxidative stress induces an impairment of ETC, leading to further ROS production. This causes a reduction in mitochondrial β‐oxidation, promoting fat accumulation and resulting in hepatic steatosis. 2. Oxidative stress results in mtDNA oxidation, leading to the release of mtDNA into the cytoplasm. This activates the NLRP3 inflammasome, triggering inflammation and contributing to liver injury. Both these processes are enhanced by impaired mitophagy with accumulation of damaged mitochondria. AMPK: 5′‐prime‐AMP‐activated protein kinase; SIRT1: Sirtuin 1; PGC1α: PPARG Coactivator 1 Alpha; ACC: Acetyl‐CoA carboxylase; FOXO1: Forkhead box protein O1; DNL: *De novo* lipogenesis; FAO: Fatty acid oxidation; PPARα: Peroxisome Proliferator‐Activated Receptor Gamma; S100A11: S100 Calcium Binding Protein A11; ROS: Reactive Oxygen Species; ETC: Mitochondrial electron transport chain; mtDNA: Mitochondrial DNA; NLRP3: NLR Family Pyrin Domain Containing.

Imbalances between lipid uptake and synthesis on one hand, and clearance on the other, result in LD accumulation in hepatocytes, triggering cellular stress and MASLD progression [42]. Regulation of intrahepatic autophagy is key to allow LD catabolism and FAO and depends on the relative activity of key autophagy regulators, including mechanistic target of rapamycin kinase (mTORC1), a central regulator of cell growth and metabolism during nutrient excess, and 5′‐AMP‐activated protein kinase (AMPK), a cellular energy deficit sensor [40]. Specifically, mTORC1 suppresses autophagy by phosphorylating Unc‐51‐like autophagy‐activating kinase (ULK1) at Ser757 and preventing the interaction with AMPK. Conversely, AMPK: (a) promotes autophagy by activating ULK1 through phosphorylation at Ser317 and Ser777 and by inhibiting mTORC1 (Figure S1A) [40, 43]; (b) inhibits acetyl‐CoA carboxylase (ACC)‐1 and ACC‐2, reducing the cytosolic conversion of acetyl‐CoA to malonyl‐CoA, thereby suppressing *de novo* lipogenesis (DNL) and promoting FAO [44]; (c) promotes the activation of Sirtuin (SIRT)‐1, which enhances FAO by deacetylating and activating Peroxisome Proliferator‐Activated Receptor γ Coactivator 1‐Alpha (PGC1α). In turn, PGC1α acts as a coactivator of Peroxisome Proliferator‐Activated Receptor (PPAR)‐α, leading to the upregulation of FAO‐related genes regulating mitophagy and lipophagy [44, 45]. Importantly, in the context of MASLD, hepatic AMPK activity is significantly reduced, mainly due to lipid overload and increased inflammation [46, 47] thus enhancing DNL and downregulating FAO and lipophagic pathways (Figure 2A).

**FIGURE 3:**
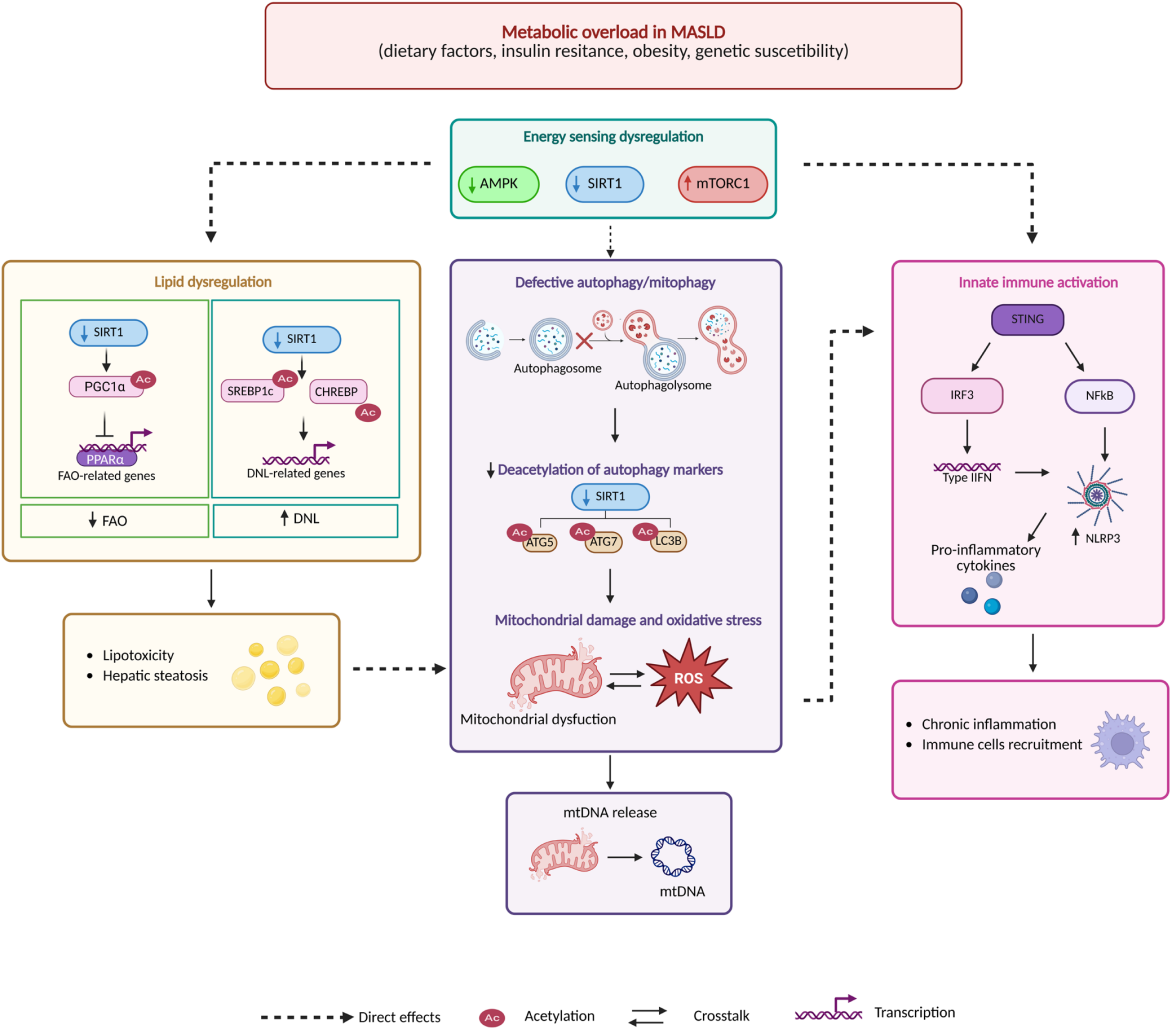
Integrated model of MASLD pathogenesis linking metabolic stress, autophagy impairment and innate immune activation. Chronic metabolic overload in MASLD disrupts the main cellular energy‐sensing pathways, characterized by mTORC1 activation and AMPK downregulation. Reduced AMPK signalling also decreases SIRT1 deacetylase activity, leading to persistent acetylation of key downstream substrates involved in autophagy regulation (ATG proteins and LC3B), fatty acid oxidation (PGC1α/PPARα axis), and *de novo* lipogenesis (SREBP1 and ChREBP pathways). These alterations collectively promote lipid accumulation and lipotoxicity. Impaired autophagy and mitophagy further exacerbate mitochondrial dysfunction, leading to ROS generation and mtDNA release, which activate innate immune pathways, including the STING–IRF3/NFκB axis and the NLRP3 inflammasome. The resulting inflammatory mediators amplify oxidative stress and hepatocellular injury through feed‐forward mechanisms, thereby driving the progression from steatosis to steatohepatitis, fibrosis and advanced liver disease. AMPK, 5′‐prime‐AMP‐activated protein kinase; ATG, autophagy‐related gene; ChREBP, carbohydrate‐responsive element‐binding protein; DNL, *De novo* lipogenesis; FAO, fatty acid oxidation; IFN, interferon; IRF3, interferon regulatory factor 3; LC3B, microtubule‐associated protein 1 light chain 3 B; MASLD, metabolic dysfunction‐associated steatotic liver disease; mtDNA, mitochondrial DNA; mTORC, mechanistic target of rapamycin kinase; NFᴋB, nuclear factor kappa B; NLRP3, NLR family pyrin domain containing; PGC1α, PPARG coactivator 1 alpha; PPARα, peroxisome proliferator‐activated receptor gamma; ROS, reactive oxygen species; SIRT1, sirtuin 1; SREBP, sterol regulatory element binding transcription factor 2; STING, synthase‐stimulator of interferon genes.

SIRT1, a directing sensor of intracellular energy deficit, is another key inducer of autophagy through the deacetylation of ATG5, ATG7, and LC3B (Figure 3) [48]. Overall, SIRT1 has emerged as a critical modulator of MASLD progression by promoting FAO, reducing lipogenesis, activating ATGL and restraining fibrogenesis [49]. Additionally, SIRT1 exerts metabolic control by deacetylating and rendering transcriptionally active Forkhead box protein O1 (FOXO1), thereby promoting lipophagy and counteracting ROS accumulation [50]. Because of MASLD‐dependent AMPK downregulation, SIRT1 activity is also lowered, compromising its protective role (Figures 2A and 3).

Non‐canonical lipophagic mechanisms have also recently been implicated in MASLD. Indeed, the S100A11 protein has emerged as a novel regulator of hepatic lipid metabolism, being overexpressed in both in vitro and in vivo MASLD models as well as in patients. Interestingly, S100A11 upregulation inhibits histone deacetylase 6 (HDAC6), leading to FOXO1 hyper‐acetylation, which impairs its canonical pro‐lipophagic transcriptional activity, triggering the expression of lipogenic genes, including Cell Death Inducing DFFA Like Effector C (CIDEC) and Diacylglycerol O‐Acyltransferase (DGAT)‐2. Functional studies further demonstrated that inhibiting FOXO1 or deleting DGAT2 prevents hepatic lipid accumulation [51]. These findings reveal a novel S100A11–HDAC6–FOXO1 axis linking lipogenesis to MASLD via non‐canonical autophagy pathways, suggesting that autophagy‐related proteins are not only implicated in LD degradation but also in the regulation of LD expansion and remodelling under metabolic stress (Figure 2A). Indeed, a paradigm‐changing study revealed that LC3 lipidation drives starvation‐induced LD biogenesis, while autophagy inhibition reduces TAG‐rich LD formation [52]. More recently, researchers observed LC3B conjugated directly onto large LDs in starved adipocytes and hepatocytes—a process mediated by ATG3—highlighting a non‐canonical autophagy mechanism that supports LD formation [53]. Therefore, the S100A11‐FOXO1 pathway could serve as a therapeutic target for modulating lipid accumulation and MASLD progression [51, 54, 55].

## 4 Mitophagy Dysfunction in MASLD

Over the past six years, mitophagy has emerged as a key determinant of MASLD progression. Elevated ROS impairs the mitochondrial electron transport chain (ETC), leading to a vicious cycle of ROS production, accumulation of malfunctioning mitochondria and eventually reduced FAO, which promotes hepatic lipid accumulation (Figure 2B) [56]. Key mitophagy receptors, including Parkin, PINK1, BCL2 Interacting Protein (BNIP)‐3 and FUN14 Domain Containing (FUNDC)‐1, have been identified as central mediators in MASLD, maintaining mitochondrial homeostasis and preventing disease progression [57]. Impaired mitophagy further exacerbates lipid accumulation and insulin resistance, accelerating MASLD progression in in vivo models [58]. In vitro models demonstrated that inhibition of mitophagy leads to the accumulation of dysfunctional mitochondria, increasing oxidative stress and inflammation. Notably, Didymin treatment of alpha mouse liver (AML)‐12 cells alleviated MASLD by activating SIRT1–dependent mitochondrial biogenesis, further highlighting SIRT1 as a therapeutic target [44, 59]. Conversely, Rho associated coiled‐coil containing protein kinase (ROCK)‐2 inhibition induces PINK1–Parkin–dependent mitophagy in in vitro hepatocytes, thereby providing protective benefits, including reduction of intracellular lipid accumulation and inflammation [60]. Notably, an increased mitochondrial DNA (mtDNA) mutation rate was detected in the D‐loop region in people with MASLD, possibly reflecting the consequences of unquenched oxidative stress and accumulation of damaged mitochondria [61]. In particular, the m.16318C>A mutation was associated with the progression of MASH, whereas the m.16129AA mutation was correlated with advanced fibrosis [61]. Additionally, the m.14766C>T missense mutation in the *mitochondrial cytochrome b* (*MT‐CYB*) gene was linked to morphological signs of damage, including the loss of mitochondrial membranes and cristae, as well as peroxisome proliferation that contribute to MASH [56]. Excess ROS oxidizes mtDNA, which is subsequently released into the cytosol, triggering an immune‐inflammatory response through activation of the NOD‐like receptor protein (NLRP)‐3 inflammasome protein, which is upregulated in the liver during MASLD [62]. Consistently, deletion of *Nlrp3* inflammasome in mice mitigates liver inflammation and fibrosis (Figure 2B) [63]. In hepatocytes, impaired mitophagy promotes cellular injury, HSC activation, and pro‐inflammatory macrophage polarization, driving MASLD and fibrosis. Conversely, restoration of mitophagy counteracts these processes by inducing apoptosis in activated HSCs and mitigating disease progression [64]. Overall, targeting the molecular mechanisms that regulate mitophagy offers promising avenues for preserving mitochondrial function and halting MASLD progression [57].

## 5 Autophagy in the Transition From Steatosis to MASH

Progression of steatosis results from elevated lipotoxicity and oxidative stress, fostering the transition to hepatocellular ballooning, cell death and inflammation and the activation of fibrogenesis, the hallmarks of MASH [65]. In the context of defective autophagy, hepatocytes are unable to efficiently clear ubiquitinated proteins and damaged cytoskeletal components, leading to the accumulation of p62/SQSTM1 and keratin aggregates. This collapse of proteostasis culminates in cytoplasmic rarefaction, keratin 8/18 disorganization, lysosomal dysfunction, and the formation of Mallory–Denk bodies (MDB)—hallmarks of ballooned hepatocytes (BH) [66, 67]. Supporting this concept, hepatocytes carrying the *ATG7* p.V471A hypomorphic variant display clear, vacuolated, or eosinophilic cytoplasm typical of BH [16, 67]. BH frequently features MDBs—cytoplasmic inclusions with a filamentous ultrastructure that appears more granular in MASH and is composed of p62/SQSTM1, keratins, and ubiquitin‐conjugated proteins [67, 68, 69, 70, 71]. In this context, the p62–kelch–like ECH–associated protein (KEAP)‐1—NFE2 Like‐BZIP‐Transcription‐Factor (NRF)‐2 (p62–KEAP1–NRF2) axis plays a pivotal role in MDBs formation and ballooning. Under physiological conditions, KEAP1 binds NRF2 and promotes its degradation. Oxidative stress disrupts this interaction, allowing NRF2 activation and the induction of antioxidant responses, while p62 facilitates KEAP1 sequestration and degradation. However, when autophagy is impaired, p62 accumulates excessively, leading to persistent and unrestrained NRF2 activation and contributing to hepatocellular damage [72, 73]. These cellular alterations, particularly the presence of MDBs and the release of damage‐associated molecular patterns (DAMPs) from degenerating BH, subsequently trigger and sustain chronic inflammation [65]. Particularly, unresolved hepatocyte cellular stress amplifies innate immune activation and establishes a pro‐inflammatory hepatic microenvironment [74], while restoration of autophagic flux appears to mitigate both oxidative stress and inflammation in HFD‐fed mice [75, 76]. Parallel to these changes, the cGAS–STING pathway has emerged as a key driver of inflammation, exhibiting bidirectional regulation with autophagy (Figure 3). Impaired autophagic flux leads to STING‐driven disruption of mitophagy and lipophagy, while STING signalling further promotes HSC activation through interactions with p62 and Neighbour of BRCA1 gene 1 (NBR1) [77, 78]. Detailed molecular crosstalk is provided in Note S1 and Figure S3. Crucially, once established, this inflammatory environment feeds back onto hepatocytes, exacerbating oxidative stress, ER stress, and further destabilizing the cytoskeleton, thereby reinforcing the cycle of injury.

Beyond hepatocytes, innate immune cells play a central role in sustaining inflammation during disease progression. Kupffer cells and monocyte‐derived macrophages (MDMs) are key regulators of the inflammatory response in MASH [79]. Notably, MDMs—the predominant macrophage population in MASH—exhibit exacerbated monocyte recruitment and hepatic inflammation when CMA is defective, thereby aggravating steatosis [80]. Consistently, macrophage‐specific *ATG5* knockout in HFD‐fed mice enhances hepatic inflammation and alters macrophage polarization, accompanied by increased IL‐1α and IL‐1β expression, thereby promoting liver injury and fibrosis progression [81, 82, 83]. Autophagy impairment also affects LSECs, promoting a pro‐inflammatory phenotype characterized by increased expression of C‐C Motif Chemokine Ligand (CCL)2, CCL5, IL6, and the Vascular Cell Adhesion Molecule (VCAM)‐1, which in turn enhances monocyte adhesion and recruitment into the hepatic parenchyma, thereby amplifying macrophage‐driven inflammation. Moreover, in LSEC lines, autophagy deficiency was associated with suppression of AMPK‐α signalling and induction of endothelial‐to‐mesenchymal transition (EMT) features, as evidenced by increased expression of alpha smooth muscle actin (α‐SMA), transforming growth factor beta (TGFβ)‐1, and collagen type I alpha 2 chain (COL1A2) [84]. These findings indicate that autophagy intrinsically restrains endothelial inflammatory activation and phenotypic transition, thereby limiting amplification of hepatic inflammation. Importantly, impaired autophagy sustains NLRP‐3 inflammasome signalling, perpetuating cytokine production and reinforcing hepatocyte injury (Figure 3) [85]. This establishes a feed‐forward loop in which hepatocyte stress promotes immune activation, and inflammatory mediators further exacerbate cellular dysfunction.

**FIGURE 4:**
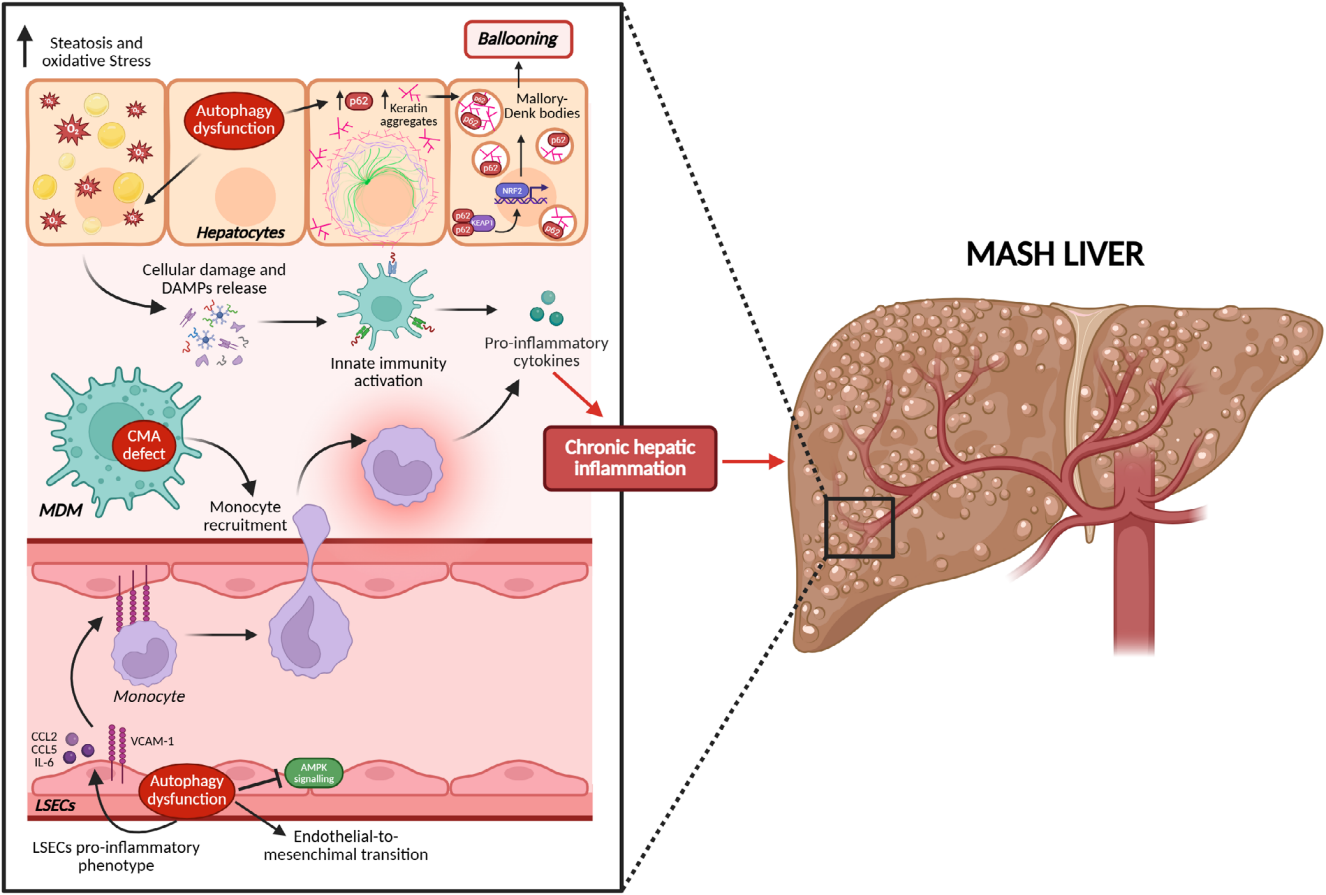
Impact of autophagy dysfunction in liver cell types on inflammation and ballooning. In hepatocytes, autophagic impairment leads to steatosis and oxidative stress, promoting the accumulation of p62 and keratin aggregates. These are subsequently sequestered into Mallory‐Denk bodies (MDBs)—a hallmark of hepatocellular ballooning—which ultimately triggers innate immunity via the release of DAMPs from damaged cells. The autophagic dysfunction also impacts the p62‐KEAP1‐NRF2 axis, contributing to hepatocellular stress. Monocyte‐derived macrophages (MDM) with defective CMA enhance monocyte recruitment to liver tissue, reinforcing the creation of a pro‐inflammatory environment. Finally, autophagy dysfunction in LSECs (i) enhances the expression of CCL2, CCL5 and VCAM‐1, facilitating monocyte recruitment and extravasation; (ii) favours the endothelial‐to‐mesenchymal transition phenotype. Overall, defective autophagy in liver cells sustains chronic inflammation, contributing to MASH development and progression. AMPK, 5′‐prime‐AMP‐activated protein kinase; CCL, C‐C Motif Chemokine Ligand; CMA, Chaperone‐mediated autophagy; DAMPs, Damage‐associated molecular patterns; IL‐6, Interleukin‐6; KEAP‐1, Kelch‐like ECH–associated protein −1; LSEC, Liver Sinusoidal Endothelial cells; MDMs, Monocyte‐derived macrophages; NRF2, NFE2 Like BZIP Transcription Factor 2; p62/SQSTM1, Sequestosome 1; VCAM‐1, Vascular Cell Adhesion Molecule‐1.

Overall, defective autophagy acts as a central amplifier of both hepatocellular injury and inflammatory signalling, mechanistically linking oxidative stress, ballooning, and MDB formation, immune activation, and endothelial dysfunction during MASLD progression (Figure 4).

## 6 Autophagy in MASLD‐HCC

Impaired autophagy is increasingly recognized as a key mechanism linking MASLD to hepatic carcinogenesis, contributing to both liver fibrosis progression and malignant transformation [86]. The role of autophagy in HCC development is still poorly characterized, but initial evidence suggests a bimodal impact autophagy on hepatic carcinogenesis: acting as a tumour suppressor during chronic liver damage and multistep accumulation of somatic mutations leading to early dysplasia, whereas as promoter of neoplastic cells survival and unstrained growth, once overt malignancy is established (Figure 1) [87].

Indeed, mouse models with reduced or liver‐specific knockout of *Atg* genes (e.g., *Beclin*, *Atg5* and *Atg7*) develop spontaneously liver tumours, suggesting that basal autophagy plays a vital role in suppressing the initiation and progression of liver cancer [88, 89, 90, 91]. The mechanism seems to involve the accumulation of p62 resulting from impaired autophagy, that promotes the development of aggressive liver tumours in transgenic mice. Indeed, mice with liver‐specific deletion of both *Atg7* and *p62/SQSTM1* exhibit reduced tumour size compared to *Atg7* deletion alone, highlighting the pivotal role of p62 in mediating the neoplastic transformation [92]. Furthermore, interferon‐gamma (IFN‐γ) inhibits cell proliferation and HCC cell growth by inducing autophagy. Blocking autophagy reverses these effects, confirming its essential role in IFN‐γ‐induced tumour suppression (Figure 1) [93]. In conclusion, autophagy acts as a tumour suppressor in HCC by maintaining cellular integrity, limiting oncogenic stress, and preserving genomic stability through pathways such as p53, while its inhibition may activate compensatory tumour‐suppressive mechanisms [94, 95, 96].

Although impaired autophagy drives liver injury and carcinogenesis, its reactivation in established tumours appears to support survival and progression (Figure 1). Under stressful conditions such as starvation, hypoxia, or metabolic stress typical of the tumour microenvironment, autophagy may provide an alternative energy source, enabling cancer cells to survive.

Among 156 patients who developed HCC, high LC3‐II expression correlated with vascular invasion, lymph node metastasis, advanced tumour stage and reduced 5‐year survival, consistent with a role of autophagy induction in promoting more aggressive liver cancer [97, 98]. Epigenetic changes have been implicated in the deregulation of autophagy. miR‐375, a strong autophagy inhibitor targeting ATG7, is consistently downregulated in HCC, leading to reduced mitophagy, thereby activating mitochondrial‐mediated apoptosis and reducing hypoxic HCC cell survival (Figure 1) [97, 98]. Additionally, evidence shows miR‐375 as a potential therapeutic target that suppresses HCC by inhibiting autophagy‐mediated survival pathways, reducing tumour growth, and enhancing chemosensitivity [99, 100].

Autophagy has also been implicated in HCC treatment resistance. Clinical and experimental studies demonstrate that sorafenib induces autophagy in HCC cells, which acts as a protective stress response allowing tumour cells to evade sorafenib cytotoxicity, thus contributing to treatment resistance [101, 102, 103]. This sorafenib‐induced autophagy involves molecular signalling pathways such as Hypoxia Inducible Factor 1 Subunit Alpha (HIF‐1α)/mTOR and interactions with the tumour microenvironment, including extracellular matrix components and hypoxia‐induced mitophagy [104, 105]. Disruption of β2‐adrenergic receptor signalling, via an autophagy‐related protein complex, stabilizes HIF‐1α and enhances drug resistance [106]. The extracellular matrix influences autophagy and sorafenib resistance. Proteins like collagen and laminin‐332, interacting with integrins, promote chemoresistance through pathways involving JUN N‐terminal Kinase (JNK) and focal adhesion kinase (FAK) [105]. Additionally, extracellular vesicles, whose content and release are influenced by autophagy, can transfer resistance traits. For instance, lncRNA‐VLDL receptor is enriched in vesicles and linked to stress responses and drug resistance [107, 108]. Consequently, autophagy upregulation upon sorafenib exposure supports tumour cell survival. Additional data are required to test whether this resistance mechanism is shared by other tyrosine kinase inhibitors (TKIs). However, the combination of autophagy inhibitors with sorafenib and other TKIs can therefore be tested as a strategy to overcome HCC chemoresistance. All in all, autophagy supports HCC cell survival, metastasis, and resistance to therapy, making it a promising target for anti‐tumour strategies in advanced HCC.

## 7 Genetic Variability Impacting on Autophagy and the Risk of MASLD

**FIGURE 5:**
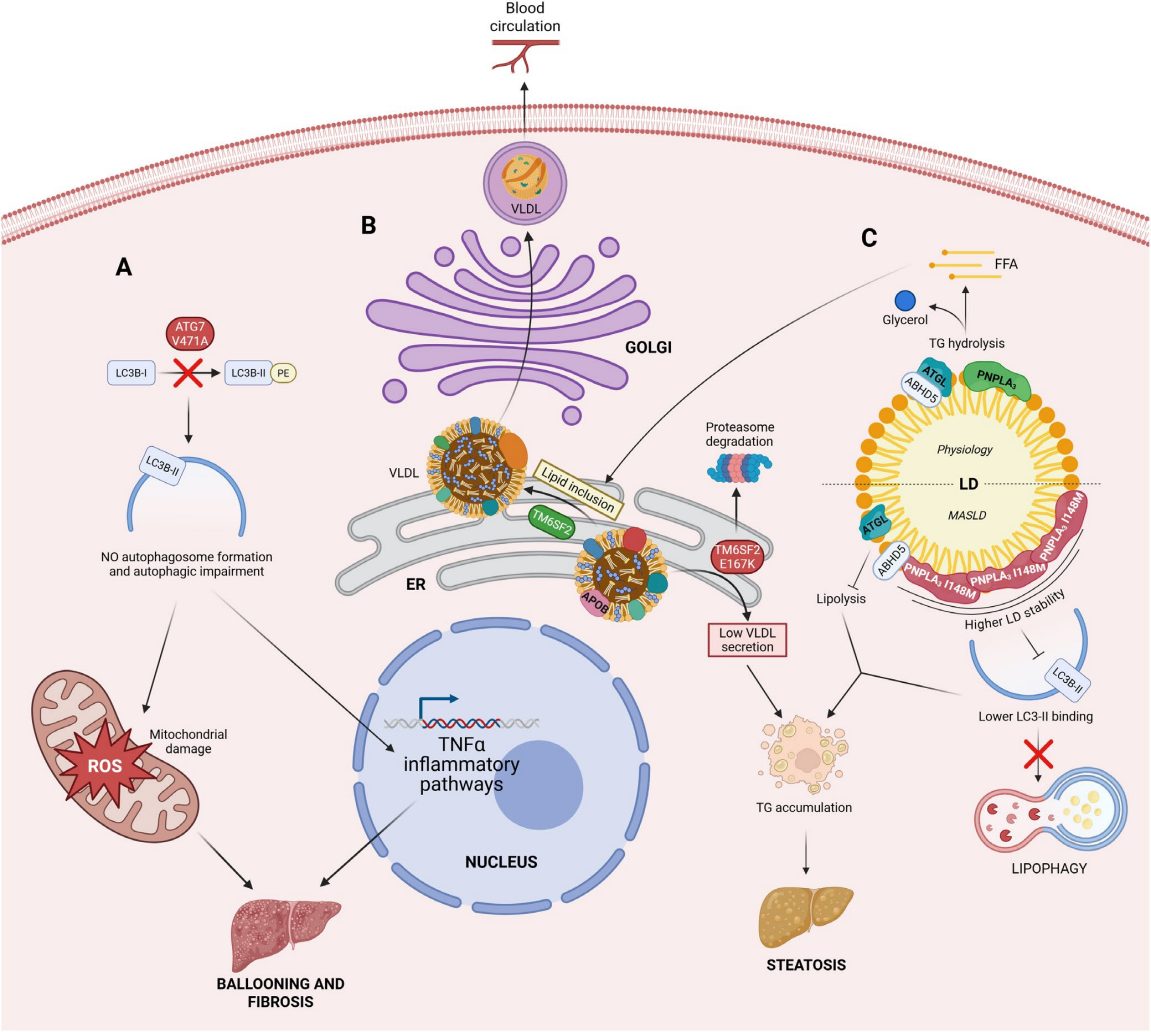
Autophagy‐related genetic variant impact on MASLD. (A) The *ATG7* p.V471A LoF variant inhibits autophagy blocking the autophagosome formation, resulting in mitochondrial damage and transcriptional upregulation of TNFα inflammatory pathways, predisposing to ballooning and fibrosis progression. (B) TM6SF2 is essential for lipid inclusion in VLDL formation in the ER: The unstable TM6SF2 E167K gets degraded and is therefore associated with lower VLDL secretion, resulting in hepatic TG accumulation and steatosis. (C) PNPLA3‐I148M protein variant has low affinity for LC3 binding, reducing lipophagy efficiency, and inhibits ATGL lipolytic activity by sequestering its coactivator ABHD5, overall resulting in hepatic TG accumulation and steatosis. ABHD5, abhydrolase domain containing 5, lysophosphatidic acid acyltransferase; APOB, apolipoprotein B100; ATG, autophagy‐related gene; ATGL, adipose triglyceride lipase; ER, endoplasmic reticulum; FFA, free fatty acids; LC3B, microtubule‐associated protein 1 light chain 3 B; LD, lipid droplet; NO, nitric oxide; PE, phosphatidylethanolamine; PNPLA3, patatin‐like phospholipase domain‐containing protein 3; ROS, reactive oxygen species; TG, triglycerides; TM6SF2, transmembrane 6 superfamily member 2; TNFα, tumour necrosis factor alpha; VLDL, very low‐density lipoproteins.

Genetic variability in autophagy‐related genes is emerging as key modulator of risk and disease progression in MASLD. Rare *ATG7* loss‐of‐function mutations, and the hypomorphic p.V471A variant carried by ~2% of the general population, impair the activation of autophagic pathways in hepatocytes and LC3‐II conversion. These variants are enriched in people with severe MASLD, especially those with obesity who progress to the development of cirrhosis and HCC [16]. Importantly, *ATG7* variants increase lipid accumulation in vitro, but are clinically associated with BH and fibrosis rather than steatosis severity [16]. Carriers of the *ATG7* p.V471A variant with histologically‐assessed MASLD had more severe ballooning scores, which was paralleled by transcriptional upregulation of inflammatory pathways and by TNFα induction in macrophage clusters in the liver lobule. In keeping, liver‐specific *Atg7* or mosaic *Atg5*/*Atg7* knockout mice develop mitochondrial swelling, accumulation of ubiquitinated proteins, elevated ROS, oxidative DNA damage, p62 buildup, and liver tumours [91]. Although direct demonstration of mitophagy defects by p.V471A in humans is still lacking, the phenotypic overlap between *ATG7*‐deficient models and patients carrying *ATG7* variants supports a mechanistic connection involving mitophagy impairment with consequent oxidative stress and tumorigenesis (Figure 5A) [91]. Thus, it is reasonable to infer that human *ATG7* variants compromise mitophagy, but functional verification (e.g., mitophagy assays in variant cells or knock‐in mice) is required. Notably, while *ATG7* variants directly impact on autophagy, common genetic variations that predispose to MASLD development and progression can also secondarily affect lipophagic processes. Indeed, inherited variants in *PNPLA3* and *TM6SF2*, accounting for the largest fraction of MASLD heritability, have a large impact on LD remodelling and regulate TAG hydrolysis and lipid secretion, resulting in disruption of lipid homeostasis and impaired lipophagy, contributing to MASLD pathogenesis [55, 109]. The key genetic determinant of MASLD is *PNPLA3*: the *PNPLA3* p.I148M variant protein localizes to larger‐sized LDs, and it has been directly linked to defective lipophagy. Indeed, the p.I148M protein variant eludes ubiquitylation and proteasome degradation, resulting in the accumulation of PNPLA3‐I148M proteins and in the impairment of triglyceride mobilization from LDs (Figure 5C) [110]. The mechanism encompasses the sequestering of the coactivator Abhydrolase Domain Containing 5, Lysophosphatidic Acid Acyltransferase (ABHD5), also known as CGI‐58, thereby reducing ATGL‐mediated lipolysis [111, 112]. Thus, PNPLA3‐I148M variant directly inhibits ATGL lipase activity, promoting TG accumulation in hepatic LDs [113]. Most importantly, the PNPLA3‐I148M variant has been reported to exhibit reduced affinity for LC3 binding, resulting in impaired recruitment to autophagosomes and selective disruption of lipophagy contributing to hepatic steatosis (Figure 5C) [114]. While the inhibition of lipophagy is expected to be milder in heterozygous individuals compared to homozygous carriers, even heterozygotes show increased risk of steatosis relative to wild‐type individuals [114]. Notably, obesity and increased body mass index (BMI) amplify the pathogenic effects of the *PNPLA3* variant [115]. Further studies are needed to elucidate the precise mechanisms by which the *PNPLA3* variant impairs lipophagy. In addition, endoplasmic reticulum (ER)‐localized TM6SF2 regulates VLDL lipidation and secretion. Dysfunctional TM6SF2 may indirectly affect autophagy through increased ER stress caused by impaired apolipoprotein B100 (ApoB100) lipidation [116, 117, 118]. The TM6SF2 p.E167K variant reduces protein stability, leading to increased hepatocellular TAG accumulation as a consequence of defective VLDL secretion and decreased ApoB100 levels (Figure 5B) [18, 119, 120, 121]. The comparison of human liver transcriptome and primary human hepatocyte spheroids between carriers and non‐carriers highlighted an upregulation of DNL, suggesting that this variant also enhances the synthesis of cholesterol or other lipotoxic lipid species [121]. These results were corroborated by Faccioli et al. [122], who showed that iPSC‐derived hepatocytes carrying *TM6SF2* p.E167K variant display mitochondrial dysfunction, oxidative stress, lipid accumulation, and impaired VLDL secretion. Notably, treatment with the chemical chaperone 4‐phenylbutyrate (4‐PBA) alleviated ER stress, reduced Sterol Regulatory Element Binding Protein 1c (SREBP‐1c)–driven lipogenesis, and attenuated the inflammatory phenotype [122]. Taken together, these data indicate that genetically driven autophagy impairment substantially contributes to hepatocellular stress and inflammation in MASLD. It remains to be conclusively proven whether decreased lipophagy, mitophagy, or a combination of both is responsible for progressive liver disease. Functional validation in human models remains a critical priority to consolidate these findings. However, the genetic architecture of MASLD does not act in isolation, as disease penetrance and severity are strongly influenced by environment–gene interactions. In particular, obesity, insulin resistance, and dietary factors synergize with genetic susceptibility to promote hepatic lipid accumulation. Furthermore, dietary fructose intake is a potent inducer of DNL, thereby exacerbating the impairment in LD turnover and lipophagy associated with *PNPLA3* and *TM6SF2* risk variants. In parallel, epigenetic mechanisms, including DNA methylation and histone modifications, may mediate the effects of environmental stimuli on metabolic gene expression, further amplifying the progression from simple steatosis to advanced fibrosis [123].

## 8 Targeting Autophagy for MASLD Therapy

**FIGURE 6:**
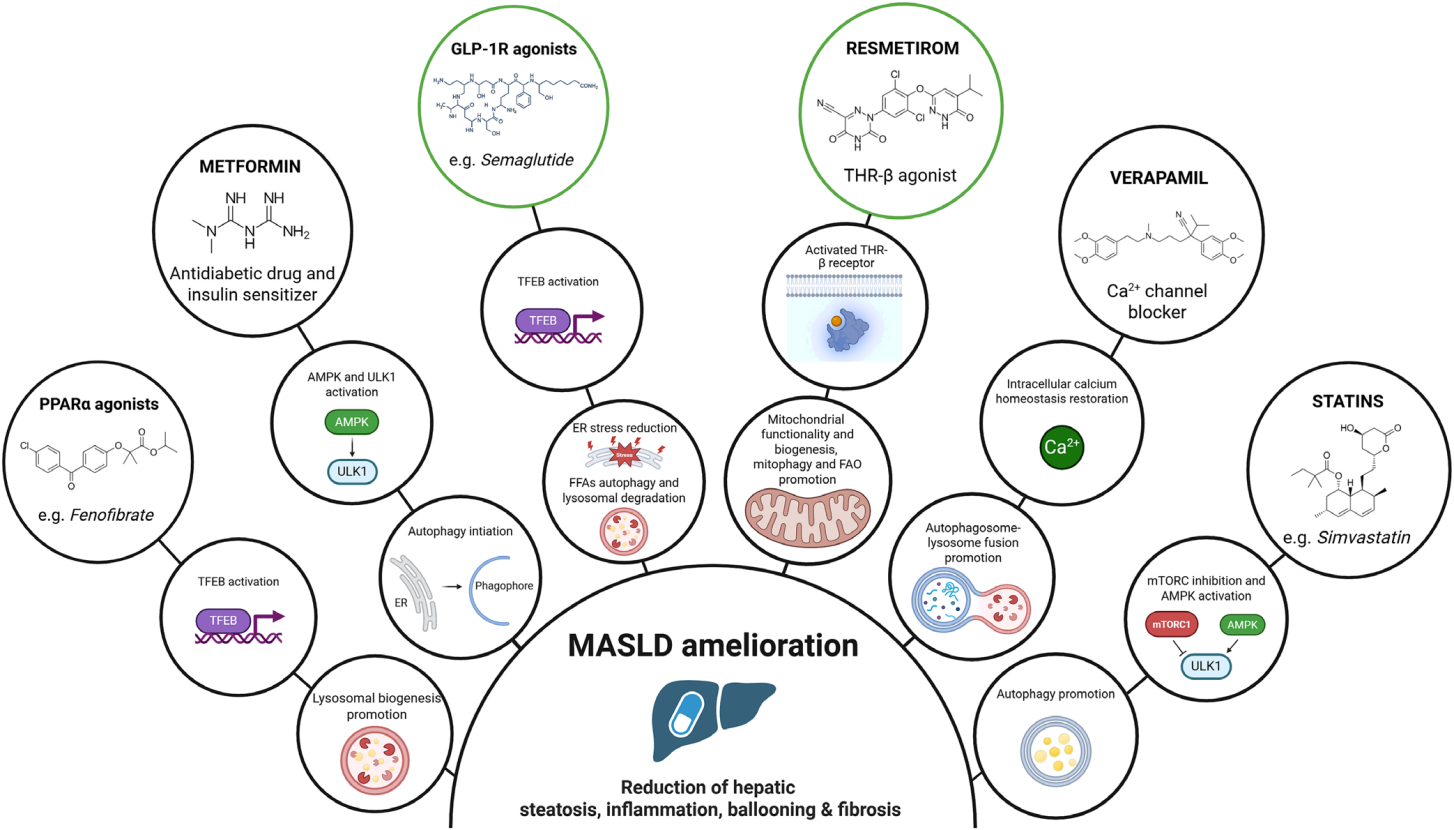
MASLD pharmacological treatment impacting on autophagy. Fenofibrate, a PPARα agonist, activates TFEB transcription factor, which promotes lysosomal biogenesis and autophagy, thereby preventing hepatic steatosis. Metformin is an antidiabetic drug and insulin sensitizer, which activates AMPK and consequently ULK1, promoting autophagy initiation and finally reducing hepatic lipid accumulation. Semaglutide is a GLP‐1 receptor agonist that reduces FFA‐induced ER stress, apoptosis, and inflammation by activating TFEB‐dependent autophagy–lysosomal lipid degradation. Another drug for MASH treatment is Resmetirom, a THR‐β agonist which enhances mitochondrial biogenesis and mitophagy, ameliorating the disease phenotype. Verapamil acts as a Ca^2+^ channel blocker, restoring calcium intracellular homeostasis and promoting autolysosome formation, thereby alleviating LDs accumulation and liver inflammation. Finally, statins, known as lipid‐lowering drugs, can also activate autophagy through mTORC1 inhibition and AMPK activation, reducing hepatic steatosis. AMPK, 5′‐prime‐AMP‐activated protein kinase; ER, endoplasmic reticulum; ER, endoplasmic reticulum; FAO, fatty acid oxidation; FDA, Food and Drug Administration; FFAs, free fatty acids; MASH, metabolic dysfunction‐associated steatohepatitis; mTORC, mechanistic target of rapamycin kinase; PPARα, peroxisome proliferator‐activated receptor gamma; TFEB, transcription factor EB; THR‐β, thyroid hormone receptor‐β; ULK1, Unc‐51‐like autophagy‐activating kinase.

Evidence is accumulating that emerging pharmacological approaches for the treatment of MASLD and the related metabolic comorbidities share a beneficial impact not only on hepatic lipid accumulation, but also on the autophagic flux in the liver (Figure 6).

Preclinical studies in murine MASLD models showed that autophagy enhancers such as carbamazepine and rapamycin effectively reduce steatosis progression [112], yet their clinical translation remains limited due to the side effects and mTOR involvement. The calcium channel blocker verapamil, which is currently approved for the treatment of hypertension and arrhythmias, restores intracellular calcium homeostasis, promoting autophagosome‐lysosome fusion, reducing LDs accumulation, and hepatic inflammation via mTOR‐independent pathways [20, 124]. Possibly through the improved autophagic flux, verapamil also enhances liver regeneration, suggesting therapeutic potential beyond lipid reduction [125].

Metformin, a first‐line antidiabetic drug, decreases hepatic glucose production primarily via the phosphorylation and activation of AMPK [126, 127]. In addition, metformin reduces hepatic inflammation by suppressing M1 macrophage polarization and modulating cholesterol metabolism in diet‐induced obese mice through a SIRT1‐dependent mechanism [128]. A meta‐analysis including six studies with 5936 patients showed that metformin improved overall 5‐year survival (OR 1.88, 95% 1.47–2.41) and decreased recurrence‐free survival rates (OR 1.83, 95% 1.40–2.40) in patients with HCC and T2D, compared to other anti‐hyperglycemic agents [129]. In keeping, a systematic review and meta‐analysis, encompassing eight observational studies (including four case–control and four cohort studies), showed that metformin use was associated with a lower risk of developing HCC compared to non‐metformin treatments. The combined OR for HCC incidence in metformin users was 0.47 (95% CI, 0.27–0.80), indicating a protective effect [130].

Some statins, including simvastatin and atorvastatin, also inhibit mTORC1 and promote autophagy in hepatocytes via AMPK activation, facilitating the degradation of LDs and thereby reducing hepatic steatosis [131]. Experimental studies using high fat diet (HFD)‐fed mouse models demonstrated that combined treatment with lovastatin and ezetimibe enhances SREBP2‐mediated autophagy, decreasing hepatic TAG accumulation [132]. Supporting these mechanistic insights, recent epidemiological data from the Rotterdam Study and PERSON cohort show that statin use was correlated with a lower prevalence of MASH and fibrosis, suggesting a protective effect against MASLD progression in humans [133]. A cross‐sectional study in clinical cohorts of patients with MASLD detected a protective and dose‐dependent relationship between statin use and lower prevalence of steatosis and fibrosis, suggesting a potential preventive effect on MASLD [133]. Interestingly, in a large cross‐sectional clinical cohort, the protective effect of statins was larger among patients negative for the *PNPLA3* p.I148M that impairs lipophagy, whereas it was absent in those homozygous for the variant [134].

Fenofibrate, a PPARα agonist, induces lipophagy by activating Transcription Factor EB (TFEB), a transcription factor that promotes lysosomal biogenesis and autophagy, thereby preventing hepatic steatosis [135].

Remarkably, resmetirom, the first thyroid hormone receptor‐β (THR‐β) agonist, became the first approved drug for fibrotic MASH. Resmetirom reduces liver fat and inflammation, induces fibrosis regression, and simultaneously improves metabolic and lipid profiles in patients [136]. Resmetirom activates thyroid hormone signalling to suppress DNL and boost lipid catabolism and directly enhances autophagy in hepatocytes. Through THR‐β, it enhances mitochondrial biogenesis, mitophagy, and β‐oxidation, indirectly promoting lipophagy [129].

Glucagon‐like protein‐1 (GLP1) receptor agonists (GLP‐1RA), and specifically semaglutide, are the second class of drugs approved for the treatment of fibrotic MASH [137]. GLP1‐RA acts primarily through the reduction of body weight and partly through improved glucose control, resulting in the amelioration of insulin sensitivity and hepatic fat accumulation [137]. However, this metabolic rewiring also translates into improved inflammation and liver fibrosis [138]. Although hepatocytes do not express canonical GLP‐1R, semaglutide may potentially reduce liver inflammation by acting on liver myeloid cells. In human hepatocytes, GLP‐1RAs decreased fatty acid‐induced ER stress and apoptosis, and enhanced lipid degradation via TFEB‐dependent activation of the autophagy–lysosomal system [139, 140], preventing the progression of MASLD into MASH [140]. Interestingly, a recent in vitro study proposed that semaglutide may improve autophagy in steatotic hepatocytes indirectly by reducing ER stress and inflammation in macrophages [141]. Additional studies are necessary to confirm this hypothesis. Moreover, GLP‐1RAs are effective in decreasing hepatic DNL by reducing the activation of carbohydrate‐responsive element‐binding protein (ChREBP), a modulator of liver lipogenesis [142]. They also decreased hepatic VLDL production by reducing the expression of SREBP‐1c and fatty acid synthase (FASN) [143]. Noteworthy, recent preclinical studies in MASH models show that semaglutide exerts antifibrotic effects not only through weight loss and reduction of hepatic steatosis but also via mechanisms independent of body weight. Notably, in a non‐obese mouse model lacking systemic metabolic dysfunction and hepatic GLP‐1R, semaglutide still improved fibrosis [144, 145].

Together, these findings underscore the therapeutic potential of targeting autophagic pathways to counteract MASLD and highlight the relevance of combining metabolic and autophagy‐modulating strategies in future clinical settings.

## 9 Personalized Risk Stratification and Therapy Selection in MASLD

Based on the evidence summarized, autophagy impairment emerges as a relevant biological feature associated with MASLD progression. Genetic variants in *ATG7* and *PNPLA3* directly impair the autophagic flux—disrupting lipophagy and mitophagy—thereby promoting hepatic lipid accumulation, inflammation, and ultimately fostering fibrosis and HCC. Notably, several of the emerging pharmacological therapies for MASLD share the ability to induce or restore autophagy through distinct mechanisms, ranging from AMPK activation (metformin and statins) to TFEB‐mediated lysosomal biogenesis (GLP‐1RA and PPARα agonists) and enhanced lipophagy and mitophagy (resmetirom). Genetic studies are key to personalized medicine, as they refine patient stratification and guide treatment choices: integrating genetic risk variants (such as those in *PNPLA3*, *TM6SF2*, *ATG7*) and circulating autophagy biomarkers (Table 1) could enable a multi‐layered stratification model for precision therapy [28, 35, 36]. The limited benefit of statins in individuals carrying the *PNPLA3* p.I148M variant highlights how genetically driven lipophagy defects can influence therapeutic responses [134]. In contrast, agents that bypass these defects—such as GLP‐1RAs, which act mainly through ER‐stress relief and TFEB pathways, or resmetirom, which enhances mitochondrial β‐oxidation and mitophagy independently of *PNPLA3*—may remain effective even in high‐risk allele carriers. Clarifying how specific variants disrupt cellular mechanisms is essential. The *ATG7* loss‐of‐function variant directly blocks autophagic flux, increasing oxidative stress [91]. This suggests that autophagy‐stimulating agents such as metformin, resmetirom, or verapamil may not be able to slow MASLD progression in patients with genetic impairment of autophagy involving ATG7, as the defect occurs at a critical step that cannot be bypassed by these treatments. In this setting, therapies targeting the resulting oxidative stress become more relevant. Antioxidant compounds—such as silibinin, which activates the SIRT1/AMPK axis and supports NAD^+^ homeostasis [146], or resveratrol, which enhances SIRT1–FOXO1 signalling and limits SREBP‐1c acetylation—may confer mechanistic advantages [147]. Likewise, vitamins E (tocopherol) and C, by reducing lipoperoxidation and mitochondrial ROS by strengthening endogenous antioxidant defences, have been shown to mitigate hepatic inflammation and fibrosis [146, 148]. Notably, in non‐diabetic patients with MASH, the PIVENS trial demonstrated that Vitamin E treatment ameliorated steatosis and lobular inflammation without worsening fibrosis [149].

Together, these interventions address the downstream redox imbalance resulting from impaired autophagy, offering a more biologically plausible strategy for individuals with genetically and environmentally driven autophagy as highlighted by polygenic and integrated risk scores.

Recent evidence showing ER stress reduction and normalization of the autophagic fluxes resulting from SREBP‐1c downregulation—achieved, for example, with 4‐PBA [122]—supports the use of GLP‐1RA as a promising therapeutic option for individuals carrying the *TM6SF2* p.E167K variant, offering an example of precision medicine in MASLD based on the integration of genetics with autophagy dynamics, outlining a new framework for personalized therapeutic decision‐making.

**FIGURE 7:**
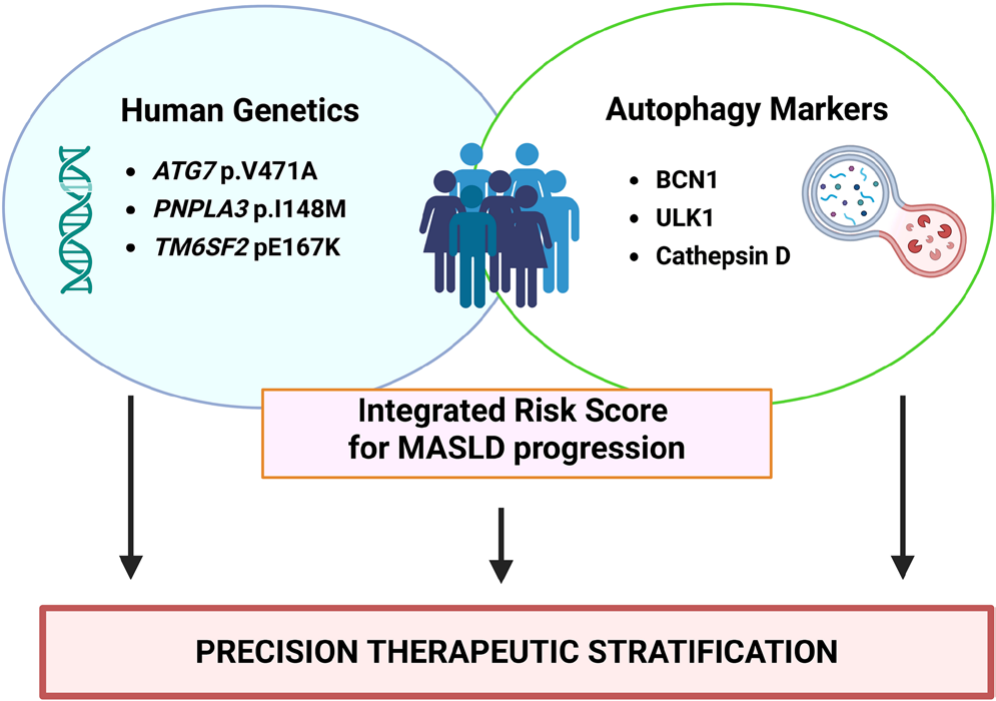
Schematic overview of personalized therapy in MASLD. Knowledge of patient‐specific genetic variants, combined with autophagy markers, could refine an integrated risk score and improve the prediction of MASLD progression. Moreover, understanding the functional impact of these variants may help identify more precise therapeutic targets for personalized treatment. Although current evidence remains limited, STING is emerging as a promising biomarker that could be integrated with genetic and autophagic data to further improve patient stratification. ATG, autophagy‐related gene; BCN1, beclin 1; cGAMP, cyclic guanosine monophosphate‐adenosine monophosphate; cGAS, cyclic GMP‐AMP; PNPLA3, patatin‐like phospholipase domain‐containing protein 3; PRS, polygenic risk score; TM6SF2, transmembrane 6 superfamily member 2; ULK1, unc‐51‐like autophagy‐activating kinase.

The integration of genetic variants with autophagy‐related biomarkers may improve the accuracy of integrated risk scores for predicting MASLD progression, facilitating early identification of high‐risk patients and more precise therapeutic stratification based on the molecular drivers of disease (Figure 7) [150].

## 10 Limitations and Future Directions

While significant strides have been made in understanding autophagy's role in MASLD and HCC, several knowledge gaps remain. The temporal dynamics of autophagic activity during disease progression are still poorly defined. Moreover, patient heterogeneity—driven by genetic factors (e.g., related to variants in *PNPLA3*, *TM6SF2*, *ATG7*), comorbidities, and environmental exposures—complicates the translation of findings into clinical practice. Future studies should focus on longitudinal human data, the validation of reliable biomarkers, and stage and MASLD subtype‐specific therapeutic interventions targeting autophagy pathways [151].

## 11 Conclusions

Autophagy has a multifaceted role in the pathophysiology of MASLD and represents a promising therapeutic target. Autophagy protects against lipid accumulation and oxidative stress, and its impairment—particularly of lipophagy and mitophagy—drives MASLD onset and progression. Genetic variants in *ATG7*, *PNPLA3*, and *TM6SF2* further exacerbate lipotoxicity and autophagy dysfunction, promoting progression to MASH fibrosis and HCCs. Integrating genetic background with autophagy‐related biomarkers, such as elevated circulating cathepsin D or reduced ULK1 and BECN1, may improve identification of risk individuals. Overall, defining autophagic defects, genetic variants, and biomarkers supports personalized therapeutic strategies targeting autophagy in MASLD.

## Author Contributions

Alessandra Cazzaniga, Alessandro Cherubini, Luca Valenti – conceptualization. Alessandra Cazzaniga, Alessandro Cherubini, Silvia Frigo – writing original draft. Alessandra Cazzaniga, Alessandro Cherubini, Eniada Rrapaj, Silvia Frigo, Luca Valenti – editing. Silvia Frigo – figure. Luca Valenti – supervision, funding acquisition.

## Funding

Italian Ministry of Health (Ministero della Salute), Ricerca Finalizzata 2021 RF‐2021‐12 373 889, Italian Ministry of Health, Ricerca Finalizzata PNRR 2022 ‘RATIONAL’ PNRR‐MAD‐2022‐12 375 656 (L.V.); Italian Ministry of Health (Ministero della Salute), Fondazione IRCCS Ca′ Granda Ospedale Maggiore Policlinico, Ricerca Corrente (L.V., D.P.). The European Union, HORIZON‐MISS‐2021‐CANCER‐02‐03 programme ‘Genial’ under grant agreement ‘101 096 312’ (L.V.); Italian Ministry of Research (MUR) PNRR—M4—C2 ‘National Center for Gene Therapy and Drugs based on RNA Technology’ CN3, Spoke 4 ‘ASSET’ (L.V.); PRIN 2022 MUR: ‘Disentangling genetic, epigenetic and hormonal regulation of Fe/heme metabolism in the gender‐specific nature of NAFLD (DEFENDER)’. Regione Lombardia FRRB From Bench to Bedside, Li*p*id d*r*oplet r*e*tention as the *c*a*u*sal d*r*iver of progre*s*sive steat*o*tic live*r* disease—hepatocellular carcinoma ‘PRECURSOR‐HCC’ (L.V.). Bando Ricerca Corrente and Piano Nazionale Complementare Ecosistema Innovativo della Salute—Hub Life Science‐Diagnostica Avanzata (HLS‐DA)—PNC‐E3‐2022‐23 683 266—‘INNOVA’. The Department of Pathophysiology and Transplantation and of Pharmacological and Biomolecular Sciences, University of Milan, are funded by the Italian Ministry of Education and Research (MUR): Dipartimenti di Eccellenza Program 2023–2027.

## Conflicts of Interest

The authors declare no conflicts of interest. L.V. reports speaking fees from: Viatris, Novo Nordisk, GSK; consulting for: Novo Nordisk, Pfizer, Boehringer Ingelheim, Resalis, Almac, AIRNA.

## Supporting information


**Figure S1:** Representative overview of autophagy pathways: (A) Macroautophagy pathway: 1. Autophagy is triggered by nutrient deprivation, which increases the levels AMPK, that, in turn, activates ULK1 through phosphorylation of Ser317 and Ser777, and decreases the levels of mTORC1 levels, that normally inhibits autophagy through phosphorylation of Ser757 on ULK1; 2. PI3K is activated and phagophore forms at ER; 3. the main autophagy regulators, i.e., LC3B‐II, are recruited and cargoes are loaded; 4. The autophagosome fuses with lysosome via SNARE complex; 5. The last step consists of cargo breakdown. (B) Microautophagy pathway is represented by the direct engulfment of cargoes in the lysosome through (i) fission‐type mechanism involving ESCRT proteins; (ii) fusion‐type mechanism involving the autophagy machinery. (C) Chaperone‐Mediated Autophagy as a selective import of proteins with KFERQ motif via HSC70 and LAMP2A receptor. AMPK, 5′‐prime‐AMP‐activated protein kinase; ULK1, Unc‐51‐like autophagy‐activating kinase; mechanistic target of rapamycin kinase (mTORC)1; PI3K, Phosphatidylinositol 3‐Kinase; PI3P, Phosphatidylinositol 3‐phosphate; ER, endoplasmic reticulum; ATG, autophagy related gene; LC3B, microtubule‐associated protein 1 light chain 3 B; PE, phosphatidylethanolamine; p62, Sequestosome‐1; Ub, ubiquitin; SNARE, soluble N‐ethylmaleimide‐sensitive factor attachment protein receptor; ESCRTS, Endosomal Sorting Complex Required for Transport; HSC70, Heat Shock Protein Family A (Hsp70) Member 8; LAMP2A, Lysosome‐associated membrane protein type 2A.
**Figure S2:** Selective types of autophagy. (A) Illustration of lipophagy mechanisms. 1. Macrolipophagy involves the degradation of LD through autophagosome formation. It can occur via two pathways: a. the Ub‐dependent pathway, PLINs are ubiquitinated and recognized by the adaptor protein p62, which binds to LC3B‐II on the forming autophagosome; b. the Ub‐independent pathway, LD‐associated lipases (HSL and ATGL) recruit the autophagic machinery (LC3B‐II) without the need for ubiquitination; 2. Microlipophagy is a direct engulfment of LDs by the lysosome, mediated by Rab7. LDs are transported to the lysosome surface, partially embedded (‘kiss and run’), and subsequently degraded inside the lysosome. (B) Illustration of two main mitophagy pathways: a. the Ub‐dependent pathway, under stress conditions, mitochondrial depolarization stabilizes PINK1 on the outer mitochondrial membrane, leading to Parkin recruitment and activation. Parkin ubiquitinates outer mitochondrial membrane proteins, which are recognized by autophagy receptors, such as p62, that bind to LC3B‐II, promoting autophagosome formation and mitochondrial degradation; b. the Ub‐independent pathway, in response to mitochondrial damage, receptors like FUNDC1 directly interact with LC3B‐II, allowing damaged mitochondria to be engulfed by autophagosomes without the need for ubiquitination. The mitochondria are then degraded following autophagosome‐lysosome fusion. ATGL, adipose triglyceride lipase; FUNDC1, FUN14 Domain Containing; HSC, Hormone‐sensitive lipase; LC3, microtubule‐associated protein 1 light chain 3 B; LD, lipid droplet; OMM, outer mitochondrial membrane; p62, Sequestosome‐1; PARL, Presenilin Associated Rhomboid Like; PINK1, PTEN Induced Kinase 1; PLNs, Periplins; Rab7, Ras‐associated GTP‐binding protein 7; TOM, translocase of the outer membrane; Ub, ubiquitin.
**Figure S3:** cGAS‐STING signalling in liver disease. The mitochondrial dysfunction and hepatocellular stress typical of MASLD provoke the extrusion of mtDNA, activating STING1 pathway and consequently the inflammatory response. STING1 also activates mTORC1, inhibiting autophagy and resulting in LDs accumulation. In hepatic macrophages, the HFD‐induced oxidative stress induces LIM domain‐containing protein‐mediated YAP nuclear translocation, activating NOTCH1 signalling: the YAP‐NOTCH1 complex enhances cGAS expression, activating the cGAS‐STING signalling and resulting in increased expression of inflammatory mediator genes. Inflammatory cytokines bring to inhibition of the autophagic flow in hepatocytes and induce HSCs activation, contributing to collagen deposition and fibrosis, in turn impacting on LSECs inhibiting angiogenesis. In HSCs, STING is normally associated with the NBR1, which inhibits the STING activation by promoting trafficking to the endosome‐lysosomal compartment for degradation. During chronic inflammation, such as in HCC liver, p62 activates STING by displacing NBR1, allowing the formation of TRIM32‐STING complex. TRIM32 ubiquitinates STING1, activating the downstream interferon cascade [Nishimura 2024]. cGAMP, Cyclic guanosine monophosphate‐adenosine monophosphate; cGAS, cyclic GMP‐AMP; FFAs, Free Fatty Acids; HCC, hepatocellular carcinoma; HFD, high‐fat diet; HSC, Hepatic Stellate Cells; IFN, Interferon; IL, Interleukin; IRF3, interferon regulatory factor 3; LD, lipid droplet; LSEC, Liver Sinusoidal Endothelial cells; mtDNA, mitochondrial DNA; mTORC, mechanistic target of rapamycin kinase; NBR1, neighbour of BRCA1 gene 1; NF‐KB, nuclear factor kappa B; NF‐KB, nuclear factor kappa B; NOTCH1, notch receptor 1; p62, Sequestosome‐1; ROS, Reactive Oxygen Species; STING, Synthase‐stimulator of Interferon Genes; TFGβ, Transforming Growth Factor‐beta; TNFα, Tumour Necrosis Factor alpha; TRIM32, Human tripartite motif family of proteins 32; Ub, ubiquitin; ULK1, Unc‐51‐like autophagy‐activating kinase; YAP, Yes‐associated protein; αSMA, alpha smooth muscle actin.

## Data Availability

Data sharing is not applicable to this article as no new data were created or analyzed during this study. All data supporting the findings of this study are available within the article and its cited references.
