# Supplementary material for: Autophagy in MASLD: A Metabolic and Precision Medicine Perspective
Source: Liver Int. 2026 Jul 29;46(9):e70816. doi: 10.1111/liv.70816 (PMC13417050; doi:10.1111/liv.70816)
Supplement: Supplementary file 1 — Figure S1: Representative overview of autophagy pathways: (A) Macroautophagy pathway: 1. Autophagy is triggered by nutrient deprivation, which increases the levels AMPK, that, in turn, activates ULK1 through phosphorylation of Ser317 and Ser777, and decreases the levels of mTORC1 levels, that normally inhibits autophagy through phosphorylation of Ser757 on ULK1; 2. PI3K is activated and phagophore forms at ER; 3. the main autophagy regulators, i.e., LC3B‐II, are recruited and cargoes are loaded; 4. The autophagosome fuses with lysosome via SNARE complex; 5. The last step consists of cargo breakdown. (B) Microautophagy pathway is represented by the direct engulfment of cargoes in the lysosome through (i) fission‐type mechanism involving ESCRT proteins; (ii) fusion‐type mechanism involving the autophagy machinery. (C) Chaperone‐Mediated Autophagy as a selective import of proteins with KFERQ motif via HSC70 and LAMP2A receptor. AMPK, 5′‐prime‐AMP‐activated protein kinase; ULK1, Unc‐51‐like autophagy‐activating kinase; mechanistic target of rapamycin kinase (mTORC)1; PI3K, Phosphatidylinositol 3‐Kinase; PI3P, Phosphatidylinositol 3‐phosphate; ER, endoplasmic reticulum; ATG, autophagy related gene; LC3B, microtubule‐associated protein 1 light chain 3 B; PE, phosphatidylethanolamine; p62, Sequestosome‐1; Ub, ubiquitin; SNARE, soluble N‐ethylmaleimide‐sensitive factor attachment protein receptor; ESCRTS, Endosomal Sorting Complex Required for Transport; HSC70, Heat Shock Protein Family A (Hsp70) Member 8; LAMP2A, Lysosome‐associated membrane protein type 2A. Figure S2: Selective types of autophagy. (A) Illustration of lipophagy mechanisms. 1. Macrolipophagy involves the degradation of LD through autophagosome formation. It can occur via two pathways: a. the Ub‐dependent pathway, PLINs are ubiquitinated and recognized by the adaptor protein p62, which binds to LC3B‐II on the forming autophagosome; b. the Ub‐independent pathway, LD‐associated lipases (HSL and ATGL) recruit t [file LIV-46-0-s001.docx]

**Autophagy in MASLD: A metabolic and precision medicine perspective**

Alessandra Cazzaniga, Silvia Frigo, Alessandro Cherubini, Eniada Rrapaj, Luca Valenti.

**SUPPLEMENTARY FILE**

**Table of context:**

**Supplementary Note:**

“Dual role of cGAS-STING: antiviral defense and driver of liver damage” page 2.

**Supplementary Figure:**

“Fig. S1. Representative overview of autophagy pathways” page 3.

“Fig. S2. Selective types of autophagy” page 5.

“Fig. S3. cGAS-STING signaling in liver disease” page 7.

**Supplementary references** page 9.

**Supplementary Note**

**Dual role of cGAS-STING: antiviral defense and driver of liver damage**

Among multiple mechanisms, dysregulation of immune signaling has been proposed to impair autophagy in MASLD, contributing to hepatocellular injury, inflammation, and fibrosis.

The cGAS-STING pathway is emerging as a potential link between innate immune signaling, autophagy regulation, and hepatic lipid metabolism ^1,2^. Under conditions of mitochondrial dysfunction and hepatocellular stress typical of MASLD, release of mtDNA into the cytosol activates cGAS-STING, triggering IFN-I responses and nuclear factor kappa B (NF-kB)-mediated inflammation, mainly through the interferon regulatory factor 3 (IRF3) ^3–5^. IRF3 acts as a transcription factor that also contributes to hepatocyte apoptosis by interacting with mitochondrial apoptotic proteins, further aggravating liver injury in MASLD ^3^. Furthermore, IRF3 contributes to the pathogenesis of insulin resistance of MASLD, through the stimulation of glucose release by hepatocytes ^6^.

The proinflammatory signaling activated by cGAS-STING exacerbates steatosis and liver injury by inducing the release of inflammatory cytokines such as Tumor Necrosis Factor (TNF)α and interleukin-6 (IL6), particularly in hepatic macrophages (**Fig. S3**) ^7,8^. Concomitantly, cGAS-STING activation disrupts the autophagic flux, including mitophagy and lipophagy, impairing cellular clearance of damaged organelles and LDs ^9,10^ and cGAS-STING promotes the activation of HSC through complex interactions involving p62/SQSTM1 and neighbor of BRCA1 gene 1 (NBR1) ^7^. Particularly, NBR1 cooperates with p62, modulating STING trafficking and degradation, impacting on inflammation and fibrosis ^4^. The cGAS–STING axis is therefore a potential link between autophagy impairment and induction of MASH, and a potential therapeutic target to counteract MASLD progression.

Conversely, activation of STING in HCC-mouse models triggered autophagy and tumor suppression. STING-deficient mice develop larger tumors at more advanced stages. Intriguingly, treatment with STING agonists restored autophagy, increased apoptosis, and enhanced IFN-I responses, reducing tumor growth ^11^. Additionally, STING activation in tumor-associated macrophages boosted antigen presentation and cytokine release, recruiting cytotoxic CD8+ T cells and shifting the tumor microenvironment towards immune activation. Overall, as for autophagy, cGAS-STING’s role in MASLD appears to be context-dependent (**Fig. S3**): while chronic activation promotes metabolic injury and inflammation, transient or controlled activation may induce protective autophagy and tumor immunity.

**Supplementary Figures.**


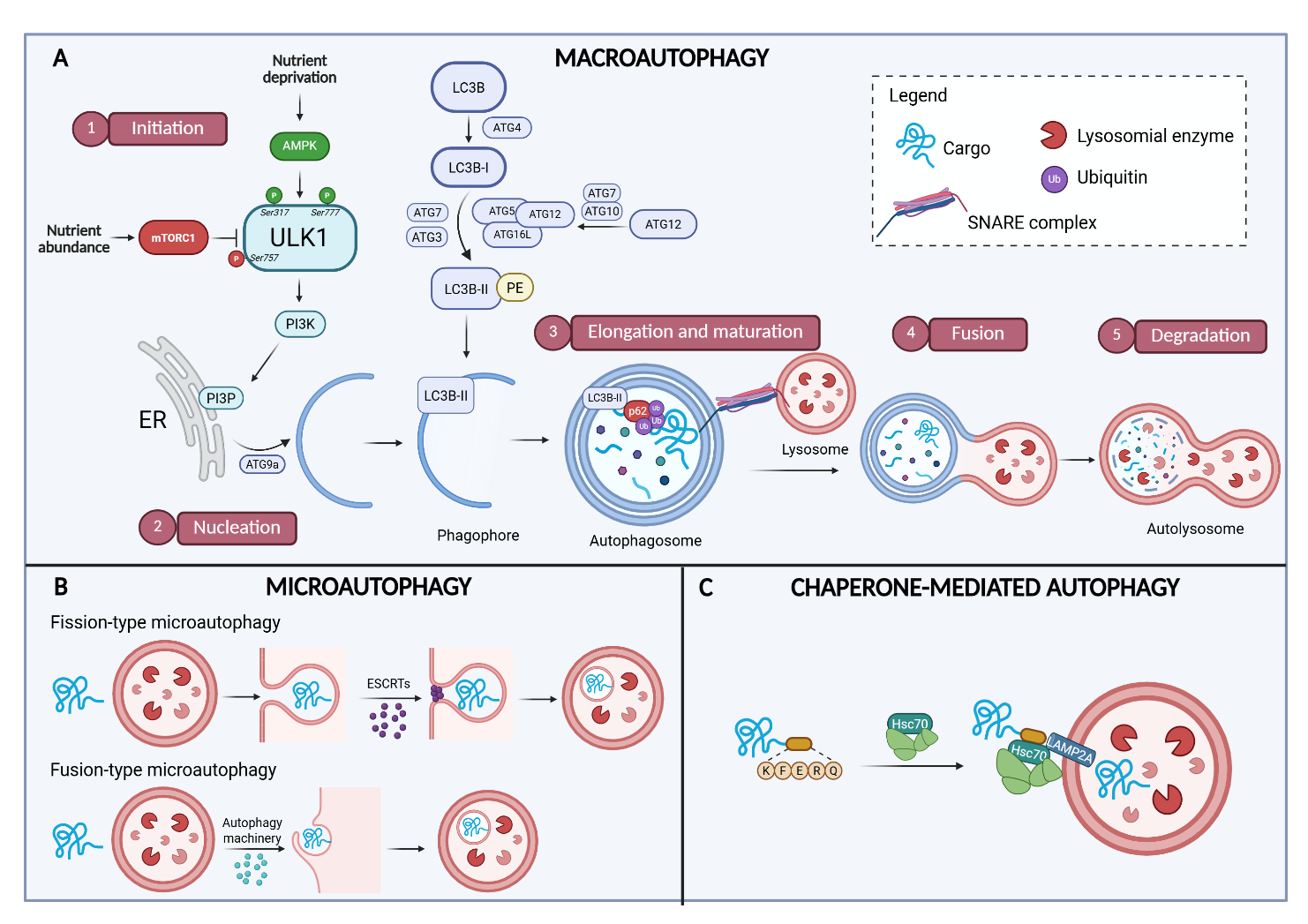


**Fig. S1. Representative overview of autophagy pathways:** (A) Macroautophagy pathway: 1. Autophagy is triggered by nutrient deprivation, which increases the levels AMPK, that, in turn, activates ULK1 through phosphorylation of Ser317 and Ser777, and decreases the levels of mTORC1 levels, that normally inhibits autophagy through phosphorylation of Ser757 on ULK1; 2. PI3K is activated and phagophore forms at ER; 3. the main autophagy regulators, i.e. LC3B-II, are recruited and cargoes are loaded; 4. The autophagosome fuses with lysosome via SNARE complex; 5. The last step consists of cargo breakdown. (B) Microautophagy pathway is represented by the direct engulfment of cargoes in the lysosome through i) fission-type mechanism involving ESCRT proteins; ii) fusion-type mechanism involving the autophagy machinery. (C) Chaperone-Mediated Autophagy as a selective import of proteins with KFERQ motif via HSC70 and LAMP2A receptor. AMPK: 5'-prime-AMP-activated protein kinase; ULK1: Unc-51-like autophagy-activating kinase; mechanistic target of rapamycin kinase (mTORC)1; PI3K: Phosphatidylinositol 3-Kinase; PI3P: Phosphatidylinositol 3-phosphate; ER: endoplasmic reticulum; ATG: autophagy related gene; LC3B: microtubule-associated protein 1 light chain 3 B; PE: phosphatidylethanolamine; p62: Sequestosome-1; Ub: ubiquitin; SNARE: soluble N-ethylmaleimide-sensitive factor attachment protein receptor; ESCRTS: Endosomal Sorting Complex Required for Transport; HSC70: Heat Shock Protein Family A (Hsp70) Member 8; LAMP2A: Lysosome-associated membrane protein type 2A.


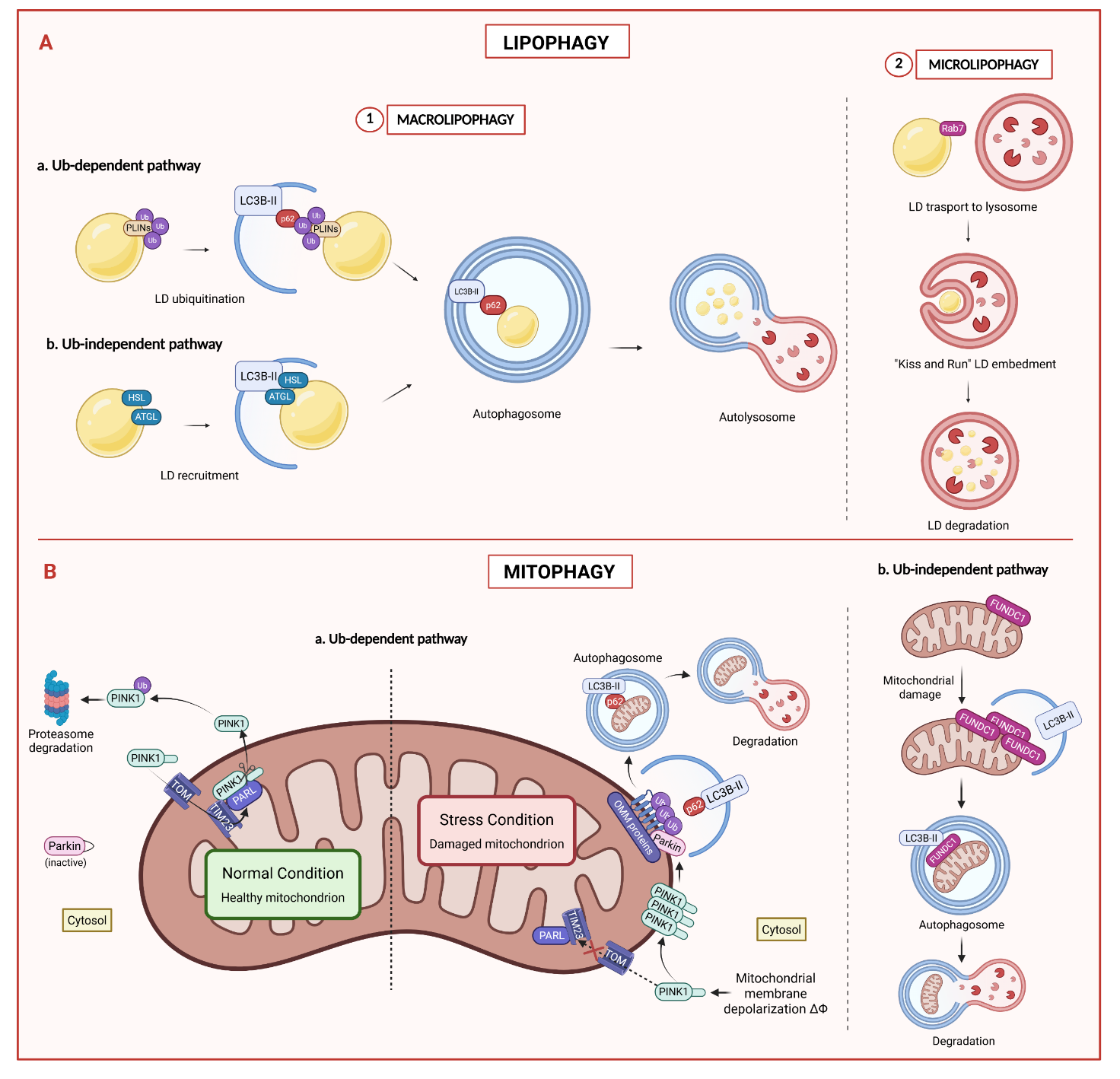


**Fig. S2. Selective types of autophagy.** (A) Illustration of lipophagy mechanisms. 1. Macrolipophagy involves the degradation of LD through autophagosome formation. It can occur via two pathways: a. the Ub-dependent pathway, PLINs are ubiquitinated and recognized by the adaptor protein p62, which binds to LC3B-II on the forming autophagosome; b. the Ub-independent pathway, LD-associated lipases (HSL and ATGL) recruit the autophagic machinery (LC3B-II) without the need for ubiquitination; 2. Microlipophagy is a direct engulfment of LDs by the lysosome, mediated by Rab7. LDs are transported to the lysosome surface, partially embedded ("kiss and run"), and subsequently degraded inside the lysosome. (B) Illustration of two main mitophagy pathways: a. the Ub-dependent pathway, under stress conditions, mitochondrial depolarization stabilizes PINK1 on the outer mitochondrial membrane, leading to Parkin recruitment and activation. Parkin ubiquitinates outer mitochondrial membrane proteins, which are recognized by autophagy receptors, such as p62, that bind to LC3B-II, promoting autophagosome formation and mitochondrial degradation; b. the Ub-independent pathway, in response to mitochondrial damage, receptors like FUNDC1 directly interact with LC3B-II, allowing damaged mitochondria to be engulfed by autophagosomes without the need for ubiquitination. The mitochondria are then degraded following autophagosome-lysosome fusion. Ub: ubiquitin; LD: lipid droplet; PLNs: Periplins; LC3: microtubule-associated protein 1 light chain 3 B; p62: Sequestosome-1; HSC: Hormone-sensitive lipase; ATGL: adipose triglyceride lipase; Rab7: Ras-associated GTP-binding protein 7; PINK1: PTEN Induced Kinase 1; TOM: translocase of the outer membrane; PARL: Presenilin Associated Rhomboid Like; OMM: outer mitochondrial membrane; FUNDC1: FUN14 Domain Containing.


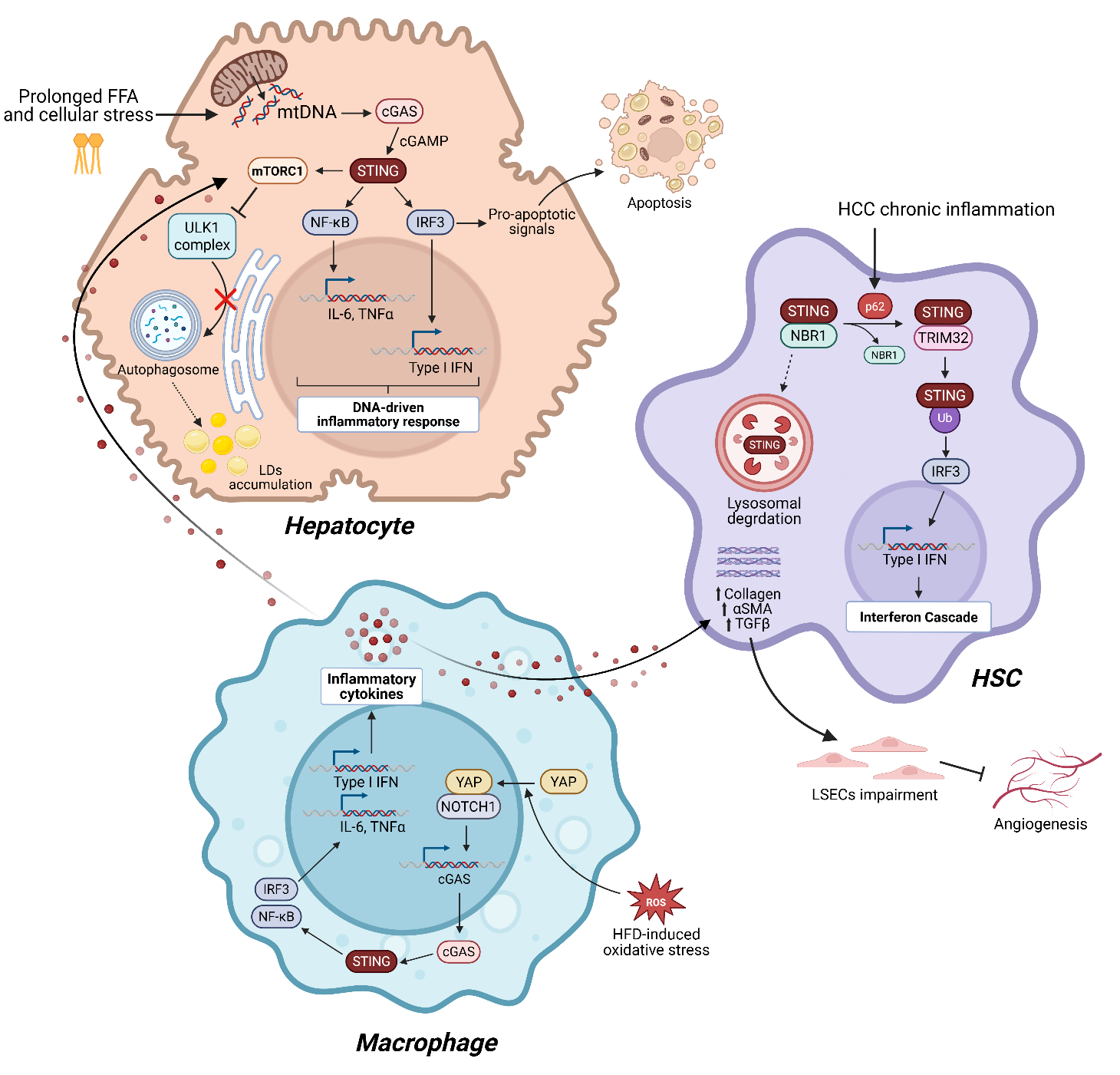


**Fig. S3**. **cGAS-STING signaling in liver disease**. The mitochondrial dysfunction and hepatocellular stress typical of MASLD provoke the extrusion of mtDNA, activating STING1 pathway and consequently the inflammatory response. STING1 also activates mTORC1, inhibiting autophagy and resulting in LDs accumulation. In hepatic macrophages, the HFD-induced oxidative stress induces LIM domain-containing protein-mediated YAP nuclear translocation, activating NOTCH1 signalling: the YAP-NOTCH1 complex enhances cGAS expression, activating the cGAS-STING signalling and resulting in increased expression of inflammatory mediator genes. Inflammatory cytokines bring to inhibition of the autophagic flow in hepatocytes and induce HSCs activation, contributing to collagen deposition and fibrosis, in turn impacting on LSECs inhibiting angiogenesis. In HSCs, STING is normally associated with the NBR1, which inhibits the STING activation by promoting trafficking to the endosome-lysosomal compartment for degradation. During chronic inflammation, such as in HCC liver, p62 activates STING by displacing NBR1, allowing the formation of TRIM32-STING complex. TRIM32 ubiquitinates STING1, activating the downstream interferon cascade [Nishimura 2024]. FFAs: Free Fatty Acids; mtDNA: mitochondrial DNA; cGAS: cyclic GMP-AMP; cGAMP: Cyclic guanosine monophosphate-adenosine monophosphate; STING: Synthase-stimulator of Interferon Genes; mTORC: mechanistic target of rapamycin kinase; ULK1: Unc-51-like autophagy-activating kinase; NF-KB: nuclear factor kappa B; IRF3: interferon regulatory factor 3; IL: Interleukin; TNFα: Tumor Necrosis Factor alpha; IFN: Interferon; LD: lipid droplet; HCC: hepatocellular carcinoma; p62: Sequestosome-1; NBR1: neighbour of BRCA1 gene 1; TRIM32: Human tripartite motif family of proteins 32; Ub: ubiquitin; αSMA: alpha smooth muscle actin; TFGβ: Transforming Growth Factor-beta; HSC: Hepatic Stellate Cells; LSEC: Liver Sinusoidal Endothelial cells; YAP: Yes-associated protein; NOTCH1: notch receptor 1; HFD: high-fat diet; NF-KB: nuclear factor kappa B; ROS: Reactive Oxygen Species.

**Supplementary references**

1. Wang JJ, Guo Y, Hu J, Peng J. STING Activation in Various Cell Types in Metabolic Dysfunction-Associated Steatotic Liver Disease. *Liver International*. 2025;45(4). doi:10.1111/liv.70063

2. Chen B, Rao X, Wang X, et al. cGAS-STING Signaling Pathway and Liver Disease: From Basic Research to Clinical Practice. *Frontiers in Pharmacology*. 2021;12. doi:10.3389/fphar.2021.719644

3. Sanz-Garcia C, McMullen MR, Chattopadhyay S, Roychowdhury S, Sen G, Nagy LE. Nontranscriptional Activity of Interferon Regulatory Factor 3 Protects Mice From High-Fat Diet-Induced Liver Injury. *Hepatology communications*. 2019;3(12):1626-1641. doi:10.1002/hep4.1441

4. Zhuang Y, Ortega-Ribera M, Thevkar Nagesh P, et al. Bile acid-induced IRF3 phosphorylation mediates cell death, inflammatory responses, and fibrosis in cholestasis-induced liver and kidney injury via regulation of ZBP1. *Hepatology : official journal of the American association for the study of liver diseases*. 2024;79(4):752-767. doi:10.1097/HEP.0000000000000611

5. Wu Y, Jin S, Liu Q, et al. Selective autophagy controls the stability of transcription factor IRF3 to balance type I interferon production and immune suppression. *Autophagy*. 2021;17(6):1379-1392. doi:10.1080/15548627.2020.1761653

6. Patel SJ, Liu N, Piaker S, et al. Hepatic IRF3 fuels dysglycemia in obesity through direct regulation of Ppp2r1b. *Sci Transl Med*. 2022;14(637):eabh3831. doi:10.1126/scitranslmed.abh3831

7. Xue F, Liu YK, Chen XY, et al. Targeting cGAS-STING: modulating the immune landscape of hepatic diseases. *Frontiers in Immunology*. 2025;16. doi:10.3389/fimmu.2025.1498323

8. Liu W, Zhang Chen Z, Yang C, et al. Update on the STING Signaling Pathway in Developing Nonalcoholic Fatty Liver Disease. *Journal of Clinical and Translational Hepatology*. 2024;12(1):91-99. doi:10.14218/JCTH.2023.00197

9. Su J, Cheng F, Yuan W. Unraveling the cGAS/STING signaling mechanism: impact on glycerolipid metabolism and diseases. *Frontiers in medicine*. 2024;11:1512916. doi:10.3389/fmed.2024.1512916

10. Wang L, Zhang Z, Zhang H, et al. The effects of cGAS-STING inhibition in liver disease, kidney disease, and cellular senescence. *Frontiers in immunology*. 2024;15:1346446. doi:10.3389/fimmu.2024.1346446

11. Thomsen MK, Skouboe MK, Boularan C, et al. The cGAS-STING pathway is a therapeutic target in a preclinical model of hepatocellular carcinoma. *Oncogene*. 2020;39(8):1652-1664. doi:10.1038/s41388-019-1108-8
